# Magnetic Hydrogels as a Treatment for Oncological Pathologies

**DOI:** 10.3390/jfb16110414

**Published:** 2025-11-05

**Authors:** Veronica Manescu (Paltanea), Adrian-Vasile Dumitru, Aurora Antoniac, Iulian Antoniac, Gheorghe Paltanea, Elena-Cristina Zeca (Berbecar), Mirela Gherghe, Iosif Vasile Nemoianu, Alexandru Streza, Costel Paun, Sebastian Gradinaru

**Affiliations:** 1 Faculty of Material Science and Engineering, National University of Science and Technology Politehnica Bucharest, 313 Splaiul Independentei, District 6, RO-060042 Bucharest, Romania; veronica.paltanea@upb.ro (V.M.); aurora.antoniac@upb.ro (A.A.); antoniac.iulian@gmail.com (I.A.); alexandru.streza@upb.ro (A.S.); 2 Faculty of Electrical Engineering, National University of Science and Technology Politehnica Bucharest, 313 Splaiul Independentei Street, District 6, RO-060042 Bucharest, Romania; 3 Department of Pathology, University Emergency Hospital, RO-030167 Bucharest, Romania; vasile.dumitru@umfcd.ro; 4 Department of Pathology, University of Medicine and Pharmacy “Carol Davila”, RO-050474 Bucharest, Romania; 5 Academy of Romanian Scientists, 54 Splaiul Independentei, RO-050094 Bucharest, Romania; 6 Estet Laser Clinique, 11 Str. Calusei, RO-030167 Bucharest, Romania; cristinazeca@gmail.com; 7 Nuclear Medicine Department, University of Medicine and Pharmacy “Carol Davila”, RO-050474 Bucharest, Romania; mirela.gherghe@umfcd.ro; 8 Nuclear Medicine Department, Institute of Oncology “Prof. Dr. Alexandru Trestioreanu”, RO-022328 Bucharest, Romania; 9 National Institute for Research and Development in Microtechnologies IMT-Bucharest, 126A Erou Iancu Nicolae Street, RO-077190 Bucharest, Romania; costel.paun@imt.ro; 10 Department of Medical-Clinical Discipline, Faculty of Medicine, Titu Maiorescu University, 67A Gheorghe Petrascu, RO-031593 Bucharest, Romania; sebastian.gradinaru@prof.utm.ro; 11 Department of General Surgery, County Hospital Ilfov, RO-050474 Bucharest, Romania

**Keywords:** magnetic hydrogel, oncology, magnetic hyperthermia, saturation magnetization, controlled drug release, magnetic field guidance, immunotherapy, cancer treatment

## Abstract

Cancer is considered today as a prevalent research direction due to the fact that, by 2050, more than 30 million cases will occur, followed by about 19 million deaths. It is expected that scholars will search for new, innovative, and localized therapies to ensure a much more targeted treatment with reduced side effects. Magnetic hydrogels overcome the disadvantages of classical magnetic nanoparticles in various oncological domains, including magnetic hyperthermia, theragnostic, immunotherapy, and, notably, regenerative medicine and contrast substances. We will review the magnetic hydrogel topics that may be involved as a potential application for cancer. Firstly, we present the international context and subject importance in the framework of statistics estimated by some researchers. Then, the magnetic hydrogel synthesis method will be briefly described with examples extracted from the literature. Supplementary, we will emphasize the main attributes of an ideal magnetic hydrogel, and last but not least, we will review some of the latest in vitro and in vivo studies in a direct relationship with magnetic hyperthermia, chemotherapeutic drug release dynamics, and immunotherapy used as single strategies or in combination, by underling the magnetic properties of the hydrogels and importance of application of magnetic fields. We will conclude our review paper by discussing toxicity issues, future trends, limitations, and proposed new approaches to address them.

## 1. Introduction

Today, oncological diseases represent one of the most important causes of death worldwide. Some statistics estimated that by 2022, about 2 million new cases, followed by 10 million deaths, will occur [1,2,3,4,5,6,7,8]. However, scholars performed an analysis and found that 33 million cases will be diagnosed by 2050, resulting in about 19 million deaths [9,10]. One can immediately notice that this is a very important issue, which is usually addressed through conventional treatments such as chemotherapy, radiotherapy, and immunotherapy. Unfortunately, these types of treatments are always accompanied by severe side effects such as nausea, blood cell modifications, digestive issues, weight changes, and even systemic organ impairment, and the potential danger of new secondary cancer occurrence [11]. In the modern world, there is a continuous search for new solutions, but this does not overcome the advantages of disease prevention and early detection [12,13,14,15].

By reviewing the literature, it can be observed that magnetic nanoparticles (MNPs) therapies are characterized by a personalized approach with reduced drawbacks, due to their local action mechanism, as well as a targeted drug delivery possibility with enhanced retention and cell membrane permeability [16,17,18,19,20]. In addition, based on the magnetic hyperthermia (MHT) effect, combining with magnetic resonance images or targeted drug delivery, a complete theragnostic approach that preserves the healthy cell integrity can be applied [21,22]. Magnetic hyperthermia could be associated with different cancer approaches, such as photothermal therapy [23] or immunotherapy [24]. At the same time, MNPs could be delivered as a magnetic solution to locally attack the cancer cell, exhibiting a specific toxicity. A detailed analysis regarding magnetic hyperthermia effects and combined treatment approaches is presented by V. Manescu (Paltanea) et al. [25] and H. Gavilan et al. [26]. One of the most important disadvantages of MNPs’ use consists of the impossibility to penetrate some tumor regions, toxicity issues, non-specific heating effects, and even the possibility to form magnetic aggregates, which lead to intense local heat at the application of an alternating magnetic field followed by intense magnetization effects [17]. Also, the retention and dispersion at the tumor site could be considered important factors to achieve a proper response of the cancerous cells [27,28,29].

To overcome the drawbacks mentioned above, MNPs are incorporated into different natural or synthetic polymeric hydrogels. In this way, nanoparticle aggregation is prohibited, and an optimal and uniform heating phenomenon occurs. In the classical case of MHT treatments, high doses of MNPs with short-term presence and quick removal, and multiple alternating magnetic field (AMF) sessions are necessary to obtain a satisfactory tumor reduction [28,30,31]. On the contrary, when a magnetic hydrogel (MG) is involved, MNPs are totally incorporated into their structure with an increased electrical conductivity, which produces adequate specific absorption rates (SAR) achievable for a reduced number of AMF radiations that respects entirely the biological limits of the product between magnetic field strength (*H*) and frequency (*f*) [32,33,34]. Also, hydrogel exhibits another advantage based on its three-dimensional structure that enhances a controlled release effect of chemotherapeutic drugs and the possibility to activate this effect under different stimuli. In addition, the toxicity related to simple MNPs’ use could be significantly reduced by the improved biocompatibility of the hydrogels. It can be foreseen that magnetic hydrogels are a very efficient tool in providing treatment for various types of cancer tumors with minimal damage to the healthy cells placed in the vicinity of the neoplasm by controlling, based on magnetic properties, the medium biochemical, mechanical, and physical characteristics [35].

**Figure 1:**
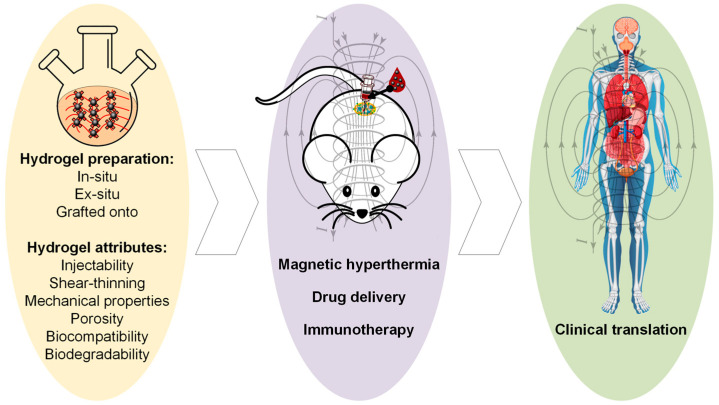
Flow-chart of the review paper, starting with magnetic hydrogel preparation methods and main attributes, in vivo investigations, and possible clinical translation. This Figure was generated using images assembled from Servier Medical Art, which are licensed under a Creative Commons Attribution 3.0 unported license (https://smart.servier.com, accessed on 27 July 2025) and www.freepik.com, accessed on 10 September 2025.

In this review paper, attention will be given to magnetic hydrogels with a possible application in cancer therapy (Figure 1). We briefly discuss the synthesis methods of MG, considering the main crosslinking strategies and the MG preparation methods, as described in preclinical cases with examples extracted from the literature. Then, the main attributes of an ideal MG used in oncology will be underlined with extended explications regarding aspects related to the most important properties, such as injectability, shear-thinning, self-healing, mechanical properties, porosity, magnetic properties, biocompatibility, and biodegradability. Last but not least, we will describe the main therapeutic routes adequate for MG, such as magnetic hyperthermia, localized drug release, and immunotherapy used as solo treatments or in different combinations. We will emphasize the importance of AMF irradiations or their absence, because some studies have proved that only the use of a particular composite material exhibiting a magnetic behavior could be enough to trigger cancer cell death. The review paper ends with future trends and challenges. In this last part, we enumerate the existing clinical trials, in which MGs’ use and information regarding the MNPs’ toxicity are involved, as well as identify some limitations and propose new future trends to address the existing literature gaps.

## 2. Synthesis Methods for Magnetic Hydrogels

One can define the hydrogels in accordance with many literature studies [35,36,37,38] as a three-dimensional pattern manufactured from hydrophilic polymers. They can absorb water and easily swell and are characterized by good elasticity, tunable physiochemistry, and high biocompatibility [39]. Usually, classical hydrogels exhibit hydrophilic functional groups, can be easily internalized by living tissue, and are suitable for theragnostic use [40]. In biomedicine, there are two types of hydrogels involved: natural ones (nucleic acids, polysaccharides—chitosan, hyaluronic acid, alginate, etc.) and synthetic ones (based on polyvinyl alcohol (PVA) or polyethylene glycol (PEG)). The advantages of natural hydrogels consist of good biocompatibility and biodegradability, but one must also consider their low mechanical properties. On the other hand, the synthetic gels have a reduced biocompatibility due to the lack of bioactivity, but exhibit adequate mechanical properties [41,42]. Through the so-called “crosslinking procedures” such as radical polymerization, physical interactions, and click chemistry [35,43], non-biodegradable or biodegradable platforms can be achieved. If we refer strictly to the magnetic hydrogel concept, this nanocomposite biomaterial could be obtained through MNPs such as iron oxide (Fe_3_O_4_ and γ-Fe_2_O_3_), transition metal ferrites (MnFe_2_O_3_, CoFe_2_O_4_) [44,45], or transition metal alloys (FePt) [46,47] that are incorporated into a polymer matrix to generate a magnetic-responsive medium.

It can be noticed that in the case of a composite biomaterial, some factors become prevalent when properties of magnetic hydrogels are discussed. Firstly, the particle size, percent of MNPs, and interactions between polymeric structure and MNPs are of utmost importance when certain magnetic properties are needed for a given medical application [48,49]. When MNPs are inserted into the hydrogel matrix, the magnetic spin moments’ rotations are prohibited, leading to a reduction of the total saturation magnetization of the entire environment. By performing different functionalization procedures, the interactions between the polymeric matrix and magnetic materials are enhanced. A direct consequence is the MNP magnetic hysteresis amplification, although attention should be given to prevent agglomerations that could occur and disturb the efficiency of the cancer treatment.

In this section, some modern procedures to obtain magnetic hydrogels will be described. We do not present in detail the classical strategies used to produce high-quality polymeric hydrogel networks, as they are extensively analyzed by Hu et al. [50] and Saini [51].

### 2.1. Crosslinking Strategies

Some of the most used techniques for preparing polymeric hydrogels are chemical crosslinking and physical crosslinking. This procedure is very important because during this step, chemical or physical bonds are correctly and strongly formed, and they lead in most cases to a three-dimensional structure with improved mechanical properties and adequate water retention [52,53]. The difference between the methods mentioned above is related to the fact that chemical crosslinking is based on strong covalent bonds, whereas physical crosslinking is characterized by weaker and sometimes reversible forces, such as ionic interactions or hydrogen bonds. One of the most important key features is that covalent bonds are directly linked to good mechanical properties; therefore, chemical crosslinking must be involved. When medical applications require improved biocompatibility or tunable properties in response to external stimuli, physical crosslinking should be the method of choice. Some controversial aspects worth mentioning include the analysis of precise action mechanisms and the establishment of an optimal balance between chemical and physical crosslinking methods when these are used in combination for different applications. To mention some “take-home” criteria, one should choose the crosslinking technology based on medical application needs (e.g., high mechanical properties—chemical crosslinking, cell-friendly and tunable property problems—physical crosslinking) and accurately evaluate the toxicity of chemical crosslinkers.

Regarding the chemical crosslinking, covalent bonds between polymeric chains occur based on different dedicated agents that exhibit functional groups [54]. Some of the most common reaction mechanisms met in practice are condensation, nucleophilic addition, and radical polymerization. Based on this procedure, chemical functionalization, gelation time, and degradation are controlled [35]. Rodkate and Rutnakornpituk [55] prepared magnetic composite microspheres with an average size of 30 μm based on radical polymerization of poly(*N*-isopropylacrylamide) in combination with magnetite nanoparticles made through the co-polymerization method and carboxymethyl chitosan, using glutaraldehyde as a crosslinking agent. The magnetic properties of the microspheres were analyzed by the vibrating sample magnetometer (VSM) quasistatic approach. It was established that their saturation magnetization (*M*_s_) was between 2.1 and 3.6 emu/g compared to 54.5 emu/g achieved for non-incorporated MNPs. Besides good magnetic properties, these biomaterials also exhibited an adequate swelling capacity and offer the possibility to release indomethacin model drug as a function of temperature and pH variation, with best theragnostic results at a temperature lower than 50 °C and a basic pH of 11.

Reversible or non-covalent bonds between polymeric chains occur during the physical crosslinking based on electrostatic interactions, van der Waals forces [54], hydrogen bonding, and hydrophobic interactions [56]. The most involved techniques are ionotropic gelation, multiple freeze–thaw cycles, and the copolymer self-assembly effect. Through physical crosslinking, modification of the hydrogel structure is usually achieved as a function of solvent chemical composition, pH, and temperature, which weakens the covalent bonds [38]. An example of application of the freezing–thawing method for magnetic hydrogels was presented by Wang et al. [57]. The authors [57] prepared magnetic chitosan/poly (vinyl alcohol) beads and tested their magnetic efficiency using the VSM technique. A moderate value for *M*_s_ of about 3.83 emu/g was achieved. The main conclusion of this study [57] was that the MNPs behaved as a cross-linker to gelated chitosan and poly (vinyl alcohol). Huang et al. [58] manufactured a magnetic nanocomposite hydrogel from poly(vinyl alcohol), magnetite Fe_2_O_3_ magnetic nanoparticles, and nano-hydroxyapatite (n-HA) based on the ultrasonic dispersion method and freeze–thaw crosslinking method. It was noticed that good mechanical properties were achieved by incorporating Fe_2_O_3_ nanoparticles and n-HA in combination with an increased biocompatibility, tested on bone marrow stromal cells (BMSCs) extracted from a New Zealand white rabbit.

As a general conclusion, the crosslinking process is of utmost importance when hydrogels with different characteristics and dedicated to various biomedical applications are involved, because these strategies could modify and adapt to the punctual medical needs, the physical and chemical properties of the composite nanomaterial.

### 2.2. Preparation Strategies for Magnetic-Based Hydrogels

The main strategies applied in the chemical practice for obtaining magnetic hydrogels can be classified as follows: in situ methods, in which MNPs are included at the same time with hydrogel synthesis, ex situ methods characterized by the fact that firstly MNPs are produced and then inserted into the hydrogel, and grafted onto methods that consist of MNPs’ functionalization and the insertion into the already prepared hydrogel. The key criteria for preparation strategy selection are usually related to medical application, to a certain MG homogeneity necessary for various treatments, and the requirement for an increased MNPs’ retention. Regarding the in situ methods, they exhibit the advantage of being used for injectable systems in minimally invasive procedures; however, the technology can be challenging to generate a homogeneous MNP distribution, leading to poor control in external magnetic fields. The grafting onto strategy leads to stable and strong anchoring of MNPs with long-term stability of magnetic properties and minimal nanoparticle loss. However, it is worth noting that this method is complex, and the shape and size of the MNPs are limited due to grafting chemistry reasons. The field has learned that injectable solutions are more effective when the in situ method is applied. The homogeneous distribution of MNPs is a measure of MG performance, and grafting-on technology is strongly linked to long-term MNP retention. On the other hand, controversial issues still exist, related to finding a balance between an easy synthesis method and optimal control, as well as biological results in relation to adequate magnetic properties. Some “take-home” criteria include the fact that scholars should choose the in situ strategy when injectability is the primary property, and the ex situ method for cases in which injectability is not prevalent, rather than a good magnetic response of MG. In addition, as stated in the previous section, the best magnetic properties are obtained when crosslink procedures are used.

Regarding the in situ methods, there almost always exists a precursor solution that is comprised of a hydrogel-forming polymer and precursors of metallic salt [59,60]. The hydrogel is manufactured through crosslinking while the particles are made and immediately dispersed into the gel [61]. This process is followed by a gel formation stage consisting of submerging into an aqueous solution or ferrofluid to reach its swelling equilibrium. Then it is once again submerged in an alkaline solution to offer adequate conditions for MNPs precipitation. As advantages of this technique, one can mention the good dispersion of MNPs into the hydrogels and the homogenous size of MNPs, and as a drawback, the fact that alkali solution might damage the hydrogel’s robust network [38]. From the in situ class, we can include the so-called blending and ferric/ferrous coprecipitation into the hydrogel matrix strategies.

Regarding the blending method, a suspension of MNPs is combined with a hydrogel precursor and then gelatinized under different conditions. In many situations, MNPs were directly encapsulated into the polymeric matrix and interacted in a low amount with the hydrogel matrix. One problem identified in practice is that MNPs could leak from the hydrogel matrix when it is put in contact with biofluids. Some immobilization procedures were proposed in the literature to minimize this risk [62,63]. As a general conclusion, the blending method is easy to apply, but in some cases, a homogenous MNPs distribution in the hydrogel could be hardly achieved [64]. Some examples of magnetic hydrogels prepared through the blending method are presented in Table 1 [65,66,67,68,69,70,71,72].

Another in situ approach is based on ferric/ferrous ion coprecipitation. The iron oxide MNPs are prepared using a Fe^3+^/Fe^2+^ aqueous solution, in which a precipitation agent such as NH_3_·H_2_O is added under temperature and inert atmosphere conditions. Usually, the co-precipitation of iron oxide MNPs is made directly into the hydrogel matrix, which is used as a chemical reactor, due to its well-known swelling capabilities. However, the hydrogel should first be synthesized through a dedicated method and then introduced into Fe^3+^/Fe^2+^ aqueous solution (molar ratio 2:1) until swelling equilibrium is achieved. Then, the newly formed magnetic composite is inserted into the alkali solution and chemically interacts with the precipitation agents to form MNPs. One can immediately notice that this approach led to a uniform dispersion of MNPs into the hydrogel networks [64]. Some studies reported that anionic polymer hydrogels that contain negatively charged groups, such as COO^-^ and SO_3_^-^, could facilitate the MNPs absorption effect [73]. This interesting approach was initiated from the idea that magneto-tactic bacteria could synthesize MNPs with a very precise structure based on their nano-magnetosome vesicles, which behave as reactors, and on negatively charged proteins that make it easy for the iron ion-binding procedure [74,75,76]. Another important factor that must be considered when the structure and yield of MNPs are analyzed is the Fe^3+^ or Fe^2+^ ion concentration, the amount of precipitants, and crosslinking density present in the hydrogel network. Examples of MG synthesized based on iron-based ion coprecipitation are provided in Table 1 [77,78,79,80,81].

In the framework of the ex situ approach, the MNPs are first synthesized through different methods and then crosslinked into the hydrogel matrix. Initially, MNPs are immersed in an aqueous solution that hinders their oxidation and aggregate formation. This ferrofluid is mixed with a hydrogel solution, and then a crosslinking method is applied to generate MG [82]. The main advantage of this ex situ technology lies in the fact that crosslinking and synthesis of MNPs are separately and independently conducted. On the other hand, a non-uniform distribution of MNPs could be noticed for hydrogels, in which the crosslinking density is very reduced, and the need for MNPs stabilization is foreseen [83]. There is an interesting approach that occurs in the literature regarding the drawbacks mentioned above. Sometimes iron oxide nanoparticles can be incorporated into polymers to obtain a better dispersion coefficient [84,85,86,87]. In the case of the click reaction hydrogel manufacturing method, it is possible to incorporate MNPs through a covalent link. Many interesting literature studies are conducted in this way [88,89]. Some investigations reported MNPs placed in crosslinking sites based on the non-covalent coordination effect [90,91]. The crosslinking method is directly related to uniform dispersion and high stability of MNPs because seeping and agglomeration do not occur. After all, MNPs are blocked in the hydrogel networks. An important drawback is the increased cost of the method and the difficulty of the production route.

As stated before, MG could be manufactured with the grafting onto method, which is based on different functional groups that are located on the MNPs surface and act as micro or nano crosslinkers. Usually, MNPs generate covalent bonds with the monomer during the polymerization process. By forming a covalent link between the polymeric matrix and MNPs, their leakage from the hydrogels is prohibited [92]. The main difference from ex situ approaches is given by the fact that in the case of grafting onto method, different functional groups are grafted onto MNPs before their insertion into the hydrogel solution. Table 1 provides examples of MGs prepared based on the grafting onto approach [93,94,95,96,97].

**Figure 2:**
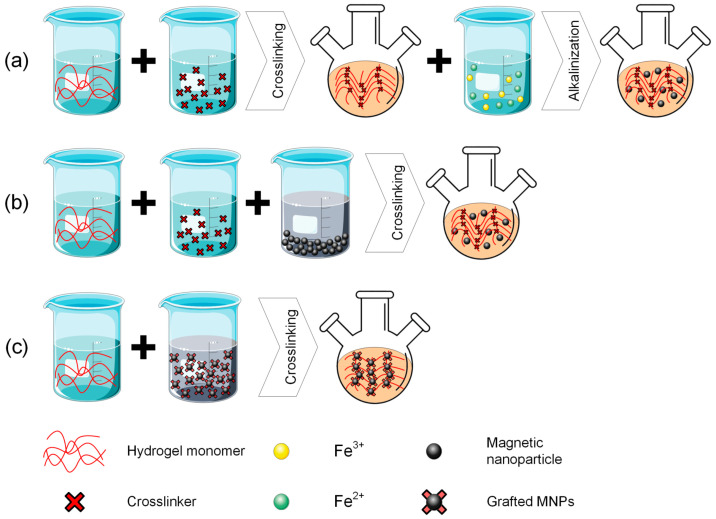
Preparation strategies for magnetic-based hydrogels: (**a**) in situ method; (**b**) ex situ method; (**c**) grafting onto method. This figure was generated using images assembled from Servier Medical Art, which are licensed under a Creative Commons Attribution 3.0 unported license (https://smart.servier.com, accessed on 27 July 2025).

As a general conclusion, one can observe that the magnetic properties of MGs are strongly influenced by the MNPs’ quantity, particle size, and position, as well as the nature of the interaction between MNPs and polymer networks. In order to improve the strength of these interactions, MNPs are functionalized with different chemical elements or groups [98]. The preparation strategies described in this section (Figure 2) should be chosen in good accordance with the medical applications, which usually require certain values for saturation magnetization of the MG and superparamagnetic behavior. The main oncological applications identified in the literature in a direct relationship with cancer disease as a possible cure are MHT, localized drug delivery, and immunotherapy. One of the most important examples of such treatments and potential applications in oncology is described together with some extracted values of magnetic parameters in Table 1.

**Table 1:** Examples of magnetic hydrogels prepared through different technologies.

Preparation Method of Magnetic Hydrogel	Polymer Type	Hydrogel Matrix	MNPs or Other Magnetic Structures/Preparation Method/MNPs’ Concentration	Magnetic Field-Related Devices, Set-Ups, and Properties	Remarks	Ref.
In situ blending method	Synthetic	Polyethylene glycol (PEG)	Ferromagnetic vortex domain iron oxide/Co-precipitation/0.6 mg/mL	10 min exposure to an alternating magnetic field (AMF) (220 Oe (17.5 kA/m), 495 kHz)/SAR of about 371 W/g (150 Oe (11.9 kA/m)) and 856 W/g (220 Oe)	Compared to control samples, which were manufactured with superparamagnetic iron oxide MNPs, almost double values of SAR were achieved	Gao et al. [63]
		Poly(N-isopropylacrylamide-*co*-acrylic acid) (poy(NIPAAm-*co*-AA))	Fe_3_O_4_ MNPs/Co-precipitation method/Fe_3_O_4_ magnetic fluid 1% molar ratio of the other components	VSM characterization/MG saturation magnetization *M*_s_ of 15 emu/g (15 Am^2^/kg) compared to 45.8 emu/g (48.5 Am^2^/kg) for pure MNPs	The prepared magnetic hydrogel exhibited superparamagnetic properties	Fan et al. [67]
	Natural/Synthetic	Alginate/polyacrylamide (PAAm) crosslinked to Fe^3+^ ions	Alginate-coated Fe_3_O_4_/Co-precipitation method/Weight percent of MNPs in ferrofluid was 1, 2, 3, 4, 5, 10, and 20 wt.% with respect to the total weight of water and polymers	VSM (735 VSM Controller, LakeShore, Westerville, OH, USA) with a maximum applied field of 9000 Oe (71.6 kA/m) at 23 °C/*M*_s_ between 18 emu/g (18 Am^2^/kg) for 20 wt.% MNPs and 1 emu/g (1 Am^2^/kg) for 1 wt.% for 1 wt.% MNPs	The prepared magnetic hydrogels exhibited outstanding mechanical properties with an increased toughness point and adequate magnetic superparamagnetic behavior	Haider et al. [62]
		Dual-network sodium alginate/poly(vinyl alcohol) PVA physically crosslinked with Ca^2+^ and freezing–thawing cycles	Magnetic laponite nanodiscs/Fe^3+^/Fe^2+^ co-precipitation in laponite/magnetic beads with 16.7 and 28.5 wt.% magnetic laponite	VSM (model 7400, LakeShore, Westerville, OH, USA)/measurements performed at ±9 kOe (71.6 kA/m) at 298 K/*M*_s_ of 2.9 emu/g (2.9 Am^2^/kg) and 13.3 emu/g (13.3 Am^2^/kg)	By adding magnetic laponite, the swelling capacity of the hydrogel decreased, but a magnetic response was achieved	Mahdavinia et al. [65]
		Chitosan/PEG	Fe_3_O_4_ MNPs/Co-precipitation/0 ÷ 40 wt.%	20 A at magnetic field strength (*H*) of 12 kA/m for 2.5 min and 15.5 A at *H* of 9.6 kA/m for another 15 min/A temperature of about 46.5 °C was achieved for 20A, while 43 °C was obtained for 15.5 A (both measurements were made for a concentration of about 30 wt.% MH)	The MH with 30 wt.% MNPs concentration was selected as an optimal platform for MHT application	Cao et al. [68]
	Natural	Collagen	Fe_3_O_4_ MNPs/-/0.5 mg/mL	By applying a constant magnetic field generated by a 255 G permanent magnet, a normal electrical activity was established in the aligned collagen fibers of MG	A new method based on gel orientation external control inside the patient’s body was proposed by directly injecting MNPs at the defect site and manipulating them based on a permanent magnet	Antman-Passig and Shefi [69]
		Agarose/collagen	Streptavidin-coated MNPs and Nano-HA-coated—γ-Fe_2_O_3_ MNPs/-/10 *v*/*v*%	By using a 2 mT cylindrical magnet (Supermagnete, Gottmadingen, Germany), a similar structure with natural multilayered tissues was obtained	The MNPs were used to align the collagen fibers in a given direction to obtain tissue-engineered structures	Betsch et al. [70]
		Type I Collagen	Magnetically oriented silica SiO_2_@Fe_3_O_4_ rods/Synthesized in the high temperature range through thermal decomposition of iron-tris(acetylacetonate) in silica rods and aligned parallel with a magnetic field based on a house-made device with two plate magnets (Nd 70 × 50 × 5 mm Ni-Cu-Ni N45)/30 mg/mL	SQUID magnetometer (Quantum Design MPMS-XL equipped with 7 T direct current (DC) magnet)/magnetic measurements performed at 2 K and zero field curves (ZFC) for a temperature range 2 K to 250 K at 100 Oe (7.9 kA/m) with temperature rate of 2 K/min/*M*_s_ of 4.5 emu/g (4.5 Am^2^/kg) obtained at 2 K from hysteresis cycle and 1.1 emu/g (1.1 Am^2^/kg) for ZFC investigation	Orientation of the magnetic nanorods exhibited an important influence on the rheological properties of MG and a moderate effect on magnetic properties	Shi et al. [66]
		Agarose	Commercial dextran-coated Micromod^®^ (Micromod, Rostock, Germany, product code 84-00-102) nanoparticles/20 wt.% (surface), 7 wt.% (middle), and 10 wt.% (deep) [71]	The magnetocompressive response of MG under the action of an external magnetic field was investigated. Magnetic field source: NdFeB magnets (0.4, 0.5, or 0.75 T; Grade N42, E-Magnets^®^, Berkhamsted, UK). By varying the MNPs’ concentration an almost linear dependence of engineering strain was achieved for agarose 1 wt.% gel, while a more constant trend was noticed in the case of 2 wt.% agarose gel	The MG behavior under the effect of an external magnetic field showed biochemical gradients and depth-dependent strain after 14 days of immersion in cell culture	Brady et al. [72]
In situ precipitation method	Synthetic	Polyethylene glycol modified with factor XIIa (PEG)	PEG-functionalized MNPs (Fe_3_O_4_ and Fe_2_O_3_)/Co-precipitation/1 mg/mL	A static magnetic field (SMF) was made by a Nd-Fe-B cylindrical magnet (thickness of 1 mm, diameter of 20 mm, magnetic induction 50 mT, Supermagnete, Uster, Switzerland). The magnetic analysis was performed in the presence of 12-well stromal vascular fraction cell plates, by placing the SMF (direction according to the north pole) below the wells. In vitro Magnetic Resonance Imaging (MRI) equipped with a Bruker Avance 300 Spectrometer (Billerica, MA, USA) were used. In the case of the SMF application, the MRI revealed different signal loss and various relaxation times	The developed magneto-mechanical actuation hydrogel proved to have a very good in vitro cell preconditioning ability	Filippi et al. [77]
		Acrylamide	Polydopamine-chelated carbon Fe_3_O_4_ nanotubes/Co-precipitation/0 ÷ 15 wt.%	Low static magnetic field (30 mT) application generated nanohybrids orientation along the SMF	A mussel-inspired approach to manufacturing a magnetic field-controlled high-conductivity hydrogel for anisotropic medical applications	Liu et al. [78]
		Six-arm star-PEG-acrylate	Superparamagnetic anionic-coated iron oxide nanoparticles (SPIONs—EMG700, Ferrotec, Unterensingen, Germany [99])/Co-precipitation/0.0046 vol%	Alignment of MG was achieved with permanent magnets with various magnetic inductions (100, 130, 300 mT)	The hierarchically designed MG exhibited the property to guide cell and nerve growth	Rose et al. [80]
	Natural/Synthetic	Alginate/PAAm	Fe_3_O_4_/Co-precipitation/concentration of Fe^2+^ and Fe^3+^ was set 0.1 M and 0.2 M, respectively	Magnetic induction heating was performed using an AMF setup (oscilloscope, water-cooling system, and induction heating system [100]). The solenoid for MHT has a diameter of 40 mm and two turns. AMF was applied for 3 min	A SAR value of 1.30 ± 0.12 W/g was achieved for 5.93 kA/m. It was concluded that the magnetic induction heating character of MG can be controlled as a function of magnetic field strength (5.93 kA/m ÷ 11.86 kA/m)	Tang et al. [73]
		Poly(*N*, *N*-dimethylacrylamide) (PDMA)/nanoclay composite	Fe_3_O_4_/Co-precipitation/14 *v*/*v*%	An induction heating system (Easyheat 224, Cheltenham Induction Heating) with a solenoid of 52 mm diameter and two turns. Saturation magnetization *M*_s_ = 10.34 emu/g (10.34 Am^2^/kg), remanent magnetization *M*_r_ = 1.33 emu/g (1.33 Am^2^/kg), coercive field *H*_c_ = 32.67 Oe (2.6 kA/m)	An intense heating process was put in evidence in combination with excellent mechanical toughness	Tang et al. [100]
	Natural	RGD peptides changed alginate	MNPs (Fe_2_O_3_)/Co-precipitation/7 wt.%	SMF application for 5 min every 12 h based on a magnet (6510 Gs (0.651 T), 1 Hz). The magnetic stimulation of the biphasic ferrogel provides adequate fatigue resistance, while the elasticity modulus and toughness were modified only to a small amount in the first 2 weeks of analysis	The biological-free MG was considered a promising candidate for muscle regeneration	Cezar et al. [79]
		Chitosan	Fe_3_O_4_/Co-precipitation/magnetite precursor containing 7 mmol Fe^3+^ and 3.5 mmol Fe^2+^	The drug release effect under a low-frequency alternating magnetic field was investigated. The drugs were loaded into 100 mL of simulated medium (phosphate-buffered solution, PBS) under the influence of a 0.4 T magnetic field generated by a rotating magnet at a frequency of 2 Hz. A total of 40% of the active drug (Adriamycin) was released in the first 2 h and was still sustained after 4 h	An improvement in the drug release phenomenon by 67.2% from the MG was obtained under magnetic field application	Wang et al. [76]
		Bacterial cellulose	Dextran-coated Fe_3_O_4_/Co-precipitation/25 ÷ 100 mM	VSM (Quantum Design, San Diego, CA, USA). Magnetization was measured at 300 K under the action of an applied magnetic field strength of ± 11,000 Oe (875.3 A/m). *M*_s_ was about 10 emu/g (10 Am^2^/kg) with no hysteresis cycle	The developed MG was dedicated to being a retention platform and cell capture for vascular tissue regeneration in the case of an intracranial aneurysm	Arias et al. [81]
Ex situ crosslinking	Synthetic	Thermosensitive P(NIPAm-*co*-Am) chelerythrine (CHE) drug loading	Fe_3_O_4_@SiO_2_ modified with vinyl groups/Solvothermal method and modified with silica by the TEOS/300 mg	VSM (Quantum Design PPMS-9, Beijing, China). The MG was characterized by zero values for coercivity and remanence with no hysteresis cycle. The saturation magnetization was estimated at 3.59 emu/g (3.59 Am^2^/kg). An AMF (5 kA/m, 100 kHz) led to about 42 °C within 30 min	The main conclusion of this study was that composite MNPs included in the hydrogel matrix resulted in an MG that could be considered for MHT applications and drug delivery	Wang et al. [87]
		Polyethylene glycol	Thiol-functionalized magnetic microparticles/−/−	Chronic magnetomechanical stimulation (4 days for 30 min/day (day 1: 0.145 μN, day 2: 0.244 μN, day 3: 0.457 μN, day 4: 1.00 μN)) was applied to modulate the expression of the human gene PIEZO2	The model presented in the paper can be used to stimulate neural types and excitable cells magnetomechanically	Tay et al. [89]
	Natural/Synthetic	Chitosan-polyolefin double network	Acid etched Fe_3_O_4_/Solvothermal method/0 ÷ 16 mg/mL	Magnetothermal properties of hydrogel were investigated under the action of AMF (282 kHz, 180 Oe (14.3 kA/m)) and showed a dose dependence variation	The developed hydrogel exhibited good mechanical properties, increased magnetothermal effects, self-healing capabilities, and high biocompatibility	Gang et al. [91]
	Natural	Dextran	Dextran-coated Fe_3_O_4_/Co-precipitation/6 wt.%	MPMS3 Quantum Design SQUID Magnetometer (Quantum Design Inc., San Diego, CA, USA). At 300 K, the magnetic hydrogel *M*_s_ was about 41.2 emu/g Fe (41.2 Am^2^/kg)	The MG exhibited good properties for using it as an MRI agent	Su et al. [85]
		Chitosan-alginate	5-Fluorouracil (Fu) loaded magnetic gelatin microspheres containing Fe_3_O_4_ MNPs/−/0.2 g	Superconducting quantum interference device magnetometer (Lake Shore 7400, Westerville, OH, USA). Hysteresis curves were measured at 300 K with a magnetic field strength of—60 ÷ 60 kOe (4.7 MA/m). Saturation was achieved at 4.95 emu/g (4.95 Am^2^/kg)	A superparamagnetic behavior for MG was evidenced in combination with good compressive and rheological properties, proving also self-healing property. The MG was considered adequate for drug delivery in cancer therapy	Chen et al. [84]
Grafting onto method	Synthetic	Polyacrylamide	Saline modified carbonyl iron (CI) particles/Silane chemistry for surface modification/-	An external magnetic field with variable magnetic flux density between 0 T and 1 T was applied, and an increase in Young’s modulus, from 10^4^ to 10^5^ Pa was achieved	The phenomenon of elastic hysteresis was observed. It was concluded that by modifying the hydrogel stiffness, various differentiations of stem cells could be achieved	Abdeen et al. [94]
	Natural/Synthetic	Hybrid polymeric material (hyaluronic acid (HA), collagen type II, and polyethylene glycol (PEG)	Poly(vinyl alcohol) modified Fe_3_O_4_	N42 neodymium magnet (D3X0, K&J Magnetics, Inc., Pipersville, PA, USA), with a height of 25 mm and a diameter of 22 mm	The MG exhibited a good biodegradation time and an increased stiffness	Zhang et al. [97]
		Methacrylate chondroitin sulfate (MA-CS) enriched with platelet lysate (PL)	MA-CS coated iron oxide MNPs/Co-precipitation/200 and 400 μg/L	Superconducting quantum interference magnetometer (SQUID-VSM, Quantum Design, San Diego, CA, USA) applied magnetic field between—20.0 and 20.0 kOe (1.6 MA/m) at room temperature. Magnetic stimulation was performed with an oscillating magnet array (2 Hz and 0.2 mm of displacement, 0.35 T/per magnet)	The magnetic field application exhibited a control effect on the hydrogel swelling. In addition, the modulation of growth factors released from platelet lysate	Silva et al. [96]
	Natural	Cellulose nanocrystal/dextran	Kartogenin (KGN) ultrasmall superparamagnetic iron oxide grafted Fe_3_O_4_@SiO_2_-KGN/Co-precipitation/0.06 ÷ 0.3 wt.%	VSM analysis. By comparing the saturation magnetization for Fe_3_O_4_ and Fe_3_O_4_@SiO_2_ -KGN, it was found in the last case that a decreased value was obtained. In addition, a superparamagnetic character was evidenced, with zero remanence and coercivity	The developed hydrogel was utilized as an MRI agent, producing high-quality images with no artifacts both in vivo and in vitro. Additionally, a long-term KGN release was evidenced	Yang et al. [93]
		Bisphosphonate (BP)—modified hyaluronic acid (HA-BP)	Fe_3_O_4_/Co-precipitation/3% (*w*/*v*)	VSM (Lake Shore 7400, Westerville, OH, USA). The MG exhibited a superparamagnetic behavior with a saturation magnetization of about 20 emu/g (1.6 MA/m) measured at 10 kOe (0.8 MA/m) applied field. To investigate the heat generation properties of MG, 1 mL of MG was subjected to the effect of AMF (528 Oe (4.2 kA/m), 258 kHz). A temperature increase of about 50 °C was reached after about 100 s of field application with a SAR value of 54.5 W/g	The novel MG that is based on dynamic coordination bonds developed between BP and MNPs used as crosslinkers resulted in a smooth, injectable, shear-thinning, and self-healing hydrogel. It was concluded that it can be successfully applied in MHT treatments	Shi et al. [90]
		Chitosan crosslinked with doxorubicin (DOX) and docetaxel (DTX) drug loading	Telechelic difunctional poly(ethylene glycol) (DF-PEG-DF) modified Fe_3_O_4_/Hydrothermal method/10 mL	SQUID MPMS XL-7 magnetometer. A saturation magnetization value of 80 emu/g (80 Am^2^/kg) was achieved at 10 kGs (1 T) magnetic flux density. The induced heating capability was analyzed under AMF (19.99 kA/m, 282 kHz) influence, and a temperature rise of 28 °C was achieved	The developed dual-drug-loaded MG could be successfully used as synergistic chemotherapy for triple-negative breast cancer	Xie et al. [88]
		Agarose	Glycosylated MNPs/−/10^11^ surface modified MNPs in 100 μL 1 wt.% agarose hydrogel	SQUID magnetometer (MPMS-7, Quantum Design, Surrey, UK). A saturation magnetization *M*_s_ of 53.6 ± 0.4 emu/g (53.6 ± 0.4 Am^2^/kg) and a magnetic susceptibility of 0.54 emu/(g·Oe) (147 m^3^/g) was obtained for glycosylated MNPs	The magnetic field influence was used for alignment of hydrogen to generate smooth biochemical gradients for cartilage tissue regeneration	Li et al. [95]

## 3. Magnetic Hydrogel Attributes for Oncological Use

In this section, the most important properties of magnetic hydrogels, which exhibit potential use in oncology for drug targeting therapy, immunotherapy, hyperthermia, or other types of treatments, will be provided. To point out adequately these attributes one should mention that the field has learned that the magnetism is an important tool and can remotely control the MGs behavior, hydrogel properties are tunable based on different MNPs concentration and various attributes such as injectability and mechanical strength can be modified, shear-thinning property is beneficial when MG are delivery trough narrow syringes, and a controlled drug release is possible if drugs are incorporated. What is still controversial in science regarding the ideal attributes of MGs is choosing an adequate crosslinking procedure, as presented before, optimizing mechanical properties, since MGs are usually fragile and brittle, and finding a scalable and cost-effective production technology. Regarding the “take-home” criteria: injectability and shear-thinning are interconnected attributes that must be optimally tuned; self-healing must be present almost always; and, last but not least, reduced toxicity should be coupled with an adequate degradation rate.

The first property that must be considered is hydrogel injectability, which is defined as the attribute of a hydrogel to be smoothly injected without high resistance into the tumor zone. This characteristic can be used in hyperthermia treatment because MG easily penetrates and blends within the irregular tissue of the tumor. It is well known that hydrogels are characterized by different viscosities, which allow their flow under the action of an externally applied pressure. When this stimulus is removed, the hydrogels retain their shape. In Ref. [101], it is stated that MG injectability represents an equilibrium state between MNPs size and distribution, hydrogel viscosity, shear-thinning property, and crosslinking density. In addition, with the help of an external magnetic field, the flow and viscosity of MG can be easily guided and controlled. Another important factor that must be considered is that MG suffers a reversible gel-sol transformation under the action of a magnetic field combined with high osmotic pressure and transit into hydrogel at 37 °C, preventing the MNPs leakage. The crosslinking density represents an important factor with high implications on both injectability and mechanical properties. If this parameter exhibits high values because of the MG’s increased stiffness, it is impossible to be injected. For reduced crosslinking density, difficult shape retention, and low mechanical properties are foreseen [102]. In addition, another property that is directly linked to MG injectability consists of shear-thinning, which could be defined as a decrease in viscosity when shear stress is applied. This property led to the facile administration of MG through injections. Table 2 provides a synthesis of some studies that describe the importance of injectability in different cancer therapies [103,104,105,106,107,108,109,110,111].

As stated before, the shear-thinning property represents a unique attribute of hydrogels, which permits the reduction of viscosity when an external shear stress load is applied and then a reversible return to the initial value when the load is no longer in action. From a physical point of view, a temporary fluidization effect could be noticed, making the hydrogels suitable for applications such as drug delivery. After the stress is removed, their consistency becomes like a classical gel, and their initial structural integrity is established. At the MNPs’ incorporation, this shear-thinning character is not highly altered. On the other hand, the obtained magnetic hydrogels can magnetically align parallel with an external magnetic field due to the superparamagnetic behavior of the MNPs [105]. By becoming magnetic responsive media, one can easily deliver different drugs only into the tumor zone [38]. Table 2 describes some examples extracted from the literature centered around the shear-thinning property [112,113,114,115,116].

Another important property of magnetic hydrogel is self-healing behavior. This is usually characterized by the material’s capacity to annihilate any damage or fracture without an external stimulus. The MNPs addition helps in a high amount of this property because they can act as crosslinking agents by bridging the near-placed polymer chains and establishing the initial structural integrity of the biomaterial [117]. At the application of an external magnetic field, the MNPs are guided and move to the defect site by generating agglomerations that facilitate new crosslinking places. It is obvious that the MNPs concentration plays an important role in the MG mesh size and swelling capacity. By increasing the MNPs concentration, the swelling degree decreases due to the reduced space between network nodes [88,118]. Some practical implementations based on the self-healing property of MG and the importance of MNPs are provided in Table 2, with a focus on cancer applications.

Mechanical properties of MGs are one of the most studied attributes for cancer applications. Attention should be devoted to MG strength, stiffness, and elasticity values. All these properties demonstrate the MG’s ability to withstand various mechanical forces in response to external stimuli. Regarding elasticity and stiffness, the polymer chemical composition and crosslinking density modulate their behavior in external magnetic fields. So, as a direct consequence, a high elasticity is directly linked to a more pronounced and faster deformation when a magnetic field is applied. In contrast, a higher mechanical stiffness could dictate a slow response to mechanical stimuli. Additionally, mechanical strength is of utmost importance because it characterizes the MG behavior when an irreversible deformation and failure occur. High elastic MGs are useful for medical applications, in which material adaptation is necessary, since an increased mechanical strength could help control, in a much more detailed manner, the drug release process in theragnostic treatments [38]. Another aspect worth mentioning is the link between hydrogel biocompatibility and its mechanical properties because mechanical attributes govern interactions between living cells and the material. Adequate values influence cell development and migration and continuously sustain the healthy cell regeneration process. Inadequate mechanical attributes usually damage the living cells by generating high inflammatory processes. Improvement of mechanical properties is performed in various ways. For instance, the use of rigid MNPs could improve the mechanical properties, as well as control the water retention and absorption [62]. Another technique consists of sacrificial bonds used for energy dissipation based on non-covalent interactions. As a result, the internal stress can be minimized in the crosslinking network, generating improved bonds after the fracture based on the self-healing characteristics described above. Table 2 provides examples of MGs with detailed mechanical property variation under external magnetic fields [62,94,119].

Another key factor that is directly related to the mechanical properties of MG is the porosity. An increased porosity reduces the stiffness and mechanical strength concomitantly with an increase in swelling and water absorption, making the MG prone to drug delivery. In an external magnetic field, the amount of MNPs is highly influenced by the pore size and shape, resulting in various behaviors and different drug kinetic release and loading capacity. In addition, high porosity exhibits adequate biological properties, which permit cell infiltration, adhesion, and proliferation, and nutrient circulation, resulting in tissue integration. On the other hand, if the porosity of the hydrogel is higher than a certain value, it could generate important mechanical instabilities and foreign body reaction at the injection site. It can be easily noticed that the precise analysis and optimization of pore structure can tailor the mechanical properties, drug release, and control in magnetic fields [38]. Table 2 shows studies that demonstrate the importance of porosity in different oncological approaches [120,121,122].

The interaction between living tissue and magnetic hydrogels could be investigated based on biocompatibility tests. These take into account the hydrogel’s adverse reaction and the MNPs’ related toxicity. In vitro analysis is based on cell type or viability-specific tests. At the same time, in vivo investigations are focused on host immune response, inflammation present in the vicinity of the injection site, as well as on some possible regeneration mechanisms. The most important attributes of MGs in a direct relationship with biocompatibility are swelling, mechanical integrity, absence of cytotoxicity, retention of magnetic properties, reduced immunogenicity, and, in some cases, the biodegradability and related by-products. Regarding the swelling behavior, it should be maintained within a certain biological limit due to induced mechanical stresses, which occur when these thresholds are overcome. In addition, the MNPs must not interact with the hydrogel swelling. The MG must maintain its structural integrity without deformation or cracks, because in this way, no secondary harmful products are emitted. MNPs should be stable, non-degradable, and have constant magnetic properties [97].

Another key factor that is usually considered in medical sciences is biodegradability. This is defined as the property of a hydrogel to break down and then be assimilated and eliminated naturally from the human body. In some cases, it is necessary to use slow degradable polymers to offer support for a longer time, but in other medical applications, rapidly degradable polymers are preferred because, in this way, supplementary free space for cell development occurs. The main mechanisms that induce the polymer breakdown are enzymatic degradation, which involves specific enzymes [123], non-enzymatic degradation based on reactive oxidative species (ROS), hydrolysis, and erosion [38]. As it was stated before, the crosslinking density and the MNPs and hydrogel chemical elements could control the degradation rate of each MG. However, in all cases, attention should be devoted to strictly evaluating the cytotoxicity of secondary products and MNPs, which must be cleared by the liver, or via the kidneys or the lymphatic system [124]. Table 2 presents studies that show the importance of biocompatibility and biodegradability of MGs.

**Figure 3:**
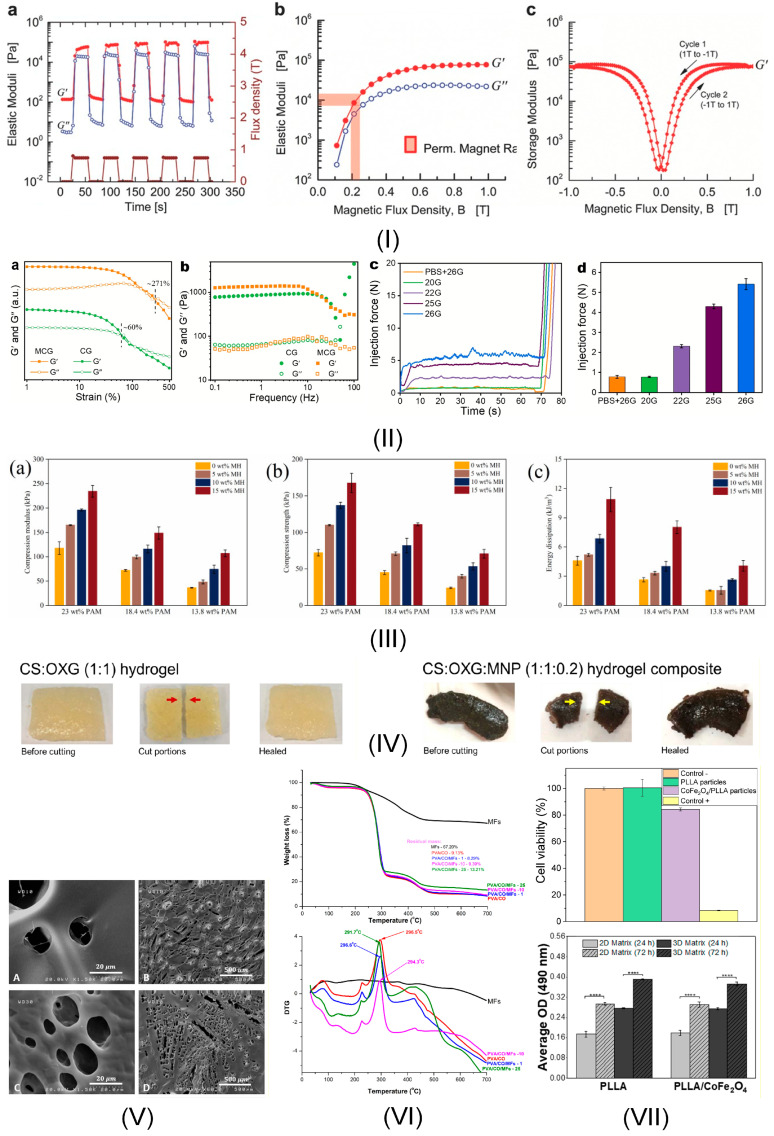
MG hydrogel attributes: (**I**) Rheological properties of polyacrylamide with carbonyl iron hydrogel: (**a**) Elastic moduli variation as a function of pulsed magnetic field from 0 to 0.75 T; (**b**) Elastic moduli versus magnetic flux density; (**c**) Storage modulus hysteresis between—1 and + 1 T. Reprinted with permission from Ref. [94]. Copyright 2025 John Wiley and Sons; (**II**) Injectability properties of montmorillonite colloidal gel with MNPs loaded with DOX: (**a**) G’ and G” variation versus strain; (**b**) G’ and G” variation versus frequency; (**c**) injection force of MG (10 *w*/*v* %) with different needles; (**d**) Injection in the first minute of administration with various needles. Reprinted from Ref. [115]; (**III**) Mechanical properties of polyacrylamide (PAM) matrix with Fe_3_O_4_ MNPs bond to the cross-linked PAM network based on hydrogen bonding: (**a**) compression modulus; (**b**) compression strength; (**c**) energy dissipation. Reprinted from Ref. [113]; (**IV**) Self-healing property of biopolymer containing oxidized xanthan gum (OXG) crosslinked with chitosan (CS) with modified magnetic Fe_3_O_4_@SiO_2_. Reprinted from Ref. [125]; (**V**) Porosity in the case of silk fibroin loaded with doxorubicin hydrochloride (DOX) and with Fe_3_O_4_ MNPs: (**A**,**B**) FESEM images of non-magnetic hydrogel; (**C**,**D**) FESEM images of magnetic hydrogel—at different magnifications. Reprinted from Ref. [121]; (**VI**) Biodegradability of poly(vinyl alcohol) (PVA)/water-soluble tricarboxy cellulose (CO)/Fe_3_O_4_ MNPs stabilized with a hydrophilic double layer of oleic acid (OA) molecules and dispersed in distilled water: TGA and DTG curves of non-magnetic and magnetic hydrogels with different MNPs concentration. Reprinted from Ref. [126]; (**VII**) Biocompatibility of poly (L-lactic acid) (PLLA) microspheres combined with magneto strictive cobalt ferrites (CoFe_2_O_4_) introduced in oil-water emulsion: cell viability of L929 fibroblasts and MC3T3-E1 pre-osteoblasts’ proliferation investigation. Reprinted from Ref. [127].

All the MGs’ attributes presented in this section must be considered when a hydrogel is intended to be used in cancer treatments. The biomaterial should be in accordance with the medical application and respect certain requirements regarding its mechanical properties, biocompatibility, biodegradability, and last but not least, injectability. There is no “gold standard” in manufacturing MGs, but as an overall impression, material property tests should be conducted in parallel with in vitro or in vivo investigations to establish the hydrogel’s suitability for oncological applications. Figure 3 presents examples extracted from the literature regarding the attributes of MG dedicated to oncological treatments.

**Table 2:** Magnetic hydrogel attributes’ importance in different cancer therapies.

Property	MG Hydrogel Characteristics	Type of Possible Application in Cancer Therapies	In Vitro/In Vivo Models	Magnetic Measurements/Devices for Magnetic Field Generation	Magnetic Field-Guided or Other Physical Mechanisms	Important Study Properties	Ref.
Injectability	Supramolecular MG self-assembled by PEGylated Fe_3_O_4_ MNPs and α-cyclodextrins (CD)	Thermo-chemotherapy (Magnetocaloric effect + MHT) for locoregional recurrence in breast cancer/MNPs were loaded with paclitaxel (PTX) or doxorubicin (DOX)	In vivo: breast mouse model (female BALB/c mice—4 weeks, 20–25 g)	VSM (LakeShore VSM 7407, Westerville, OH, USA): *M*_s_—93.9 emu/g (93.9 Am^2^/kg) at 300 K, SAR—1334 W/g Fe (heating test time 120 s) Water-cooled copper coil powered by AMF (410 kHz, 1.8 kA/m)	The drug release was accelerated due to magnetically generated heat of MNPs under AMF irradiation	Rheological parameters of MG: maximum values at 14 min and at 37 °C for storage modulus (G’) and loss modulus (G”): G’ = 6000 Pa, G” = 1000 Pa, viscosity value at 37 °C: 400 mPa·s; minimum value of viscosity at 37 °C and a shear rate of 100 (s^−1^) 2000 mPa·s	Wu et al. [103]
	Crosslinked oxidized hydroxypropyl cellulose with carboxymethyl chitosan preloaded with artesunate (ART), ferroferric oxide (Fe_3_O_4_) MNPs, black phosphorous nanosheets (BPs)	Drug delivery system with magnetic targeting, chemodynamic therapy, pH sensitivity, and photothermal therapy for eliminating hepatocellular carcinoma (HepG2) tumor	In vitro: Human HCC cell line (HepG2) and normal liver cell L02 In vivo: male BALB/c (4 weeks old)	VSM (LakeShore 7400, Westerville, OH, USA): *M*_s_—0.6 emu/g (0.6 Am^2^/kg) at 300 K Mouse anatomy-adapted 3D printed magnetic device for control of MG to the target location and tumor treatment	When the action of the magnetic field (MF) was added, it improved the targeting effect. When MF was combined with near-infrared laser irradiation (1 W/cm^2^, 5 min) tumor inhibition was achieved	Rheological parameters of MG: minimum values at 100% strain: G’ = 50 Pa, G” = 400 Pa and maximum values at a frequency of 100 Hz: G’ = 2450 Pa, G” = 200 Pa (a shear-thinning effect was noticed); minimal value of viscosity at a shear rate of 100 (Hz) was 50 Pa·s	Ma et al. [104]
	Silk fibroin and iron oxide nanocubes coated with 1,2-distearoyl-sn-glycero-3-phosphoethanolamine-N-[methoxy(polyethylene glycol)-2000] (DSPE-PEG2000)	Remote hyperthermia performance combined with thermal ablation under AMF for hepatocellular carcinoma treatment	In vitro: enzymatic degradation of MG, 4T1 cell survival analysis In vivo: MHT test on BALB/c female mice (6–8 weeks old), ultrasound-guided interventional MHT on VX-2 xenografted tumor-bearing New Zealand rabbits	SQUID (Quantum Design MPMS XL): *M*_s_—1.9 emu/g (1.9 Am^2^/kg) at 300 K and 30 kOe (2.4 MA/m). MHT test: AMF (312 kHz, 30 kA/m) Ultrasound-guided MHT: AMF (312 kHz, 30 kA/m) for 15 min	For MHT session, a magnetic field strength of 30 kA/m was applied and a temperature of 46.1 °C was reached after 3 min. AMF application, and 49.6 °C at 6 min. AMF action was achieved. For the deeper located transplantation liver tumor, when AMF with a strength of 30 kA/m was applied, a satisfactory magnetocaloric effect was considered	Rheological parameters of MG: G’ decreased with increasing shear strain (at a shear strain of 200% a value of 2.8 GPa), while the loss firstly increased to 20 Pa and then decreased to 15 Pa in the same testing conditions. It was noticed that the MG began to liquify at a shear strain of 25%	Qian et al. [105]
	Polyethylene glycol, oil-in-water, and oleic acid (OA)-coated Zn ferrite MNPs	Multimodal imaging-guided thermoablative cancer therapy (MHT)	In vivo: 4T1 xenografted tumor mice	VSM measurements at 300 K evidenced a *M*_s_ of 98.7 emu/g Fe (98.7 Am^2^/kg), higher than 56 emu/g Fe (56 Am^2^/kg) obtained for Ferumoxytol Water-cooled magnetic induction copper coil (diameter of 3 cm, length of 1.5 cm)	For in vitro magnetic heating performance analysis, an AMF (410 kHz, 1.8 kA/m) was applied for 15 min. A SAR value of 872 W/g Fe was achieved For in vivo multimodal-imaging-guided MHT, a temperature of 60 °C after 120 s AMF irradiation was noticed. After 300 s, the entire tumor was heated, and only a few degrees of increase were obtained in the neighborhood tissue	Rheological parameters of MG: maximum values at 50 °C of G’ = 22 Pa and G” = 19 Pa; viscosity at 50 °C and at 5 Hz equal to 10 Pa·s	Wu et al. [107]
	Pluronic F-127 matrix with γ-Fe_2_O_3_ MNPs loaded with DOX	Combined MHT and localized drug delivery for progressive adenocarcinoma of the ovary treatment	In vitro: OVCAR3 cell line	VSM (MDKB, Iran): *M*_s_ = 20 emu/g (20 Am^2^/kg) at room temperature and 20 kOe (1.6 MA/m)	Based on in vitro MHT tests, a temperature of about 45 °C was achieved. At the application of AMF (400 kHz, 5 min.) 75% of the DOX drug was released	Rheological parameters of MG: viscosity at 100 Hz and 10 °C (sol) was about 200 Pa·s, and at 35 °C (gel) was equal to 2700 Pa·s	Farzaneh et al. [108]
	Carboxymethylcellulose (CMC) polymer and CoFe_2_O_3_ functionalized with aminopropyl silane (NP)	Potential applications for a targeted drug delivery system	-	-	-	Rheological parameters of CMC-NP hydrogel varied after they were squeezed through a syringe from 1050 ± 140 Pa to 190 ± 20 Pa for G’, and from 80 ± 15 Pa to 20 ± 3 Pa in the case of G”	Barbucci et al. [109]
	Carboxymethylcellulose (CMC) polymer and CoFe_2_O_3_ MNPs (NP) with a concentration of 50% and 70% in relation to the polymer quantity	Drug delivery under alternating and static magnetic fields	-	-	-	Rheological parameters were determined to be a function of MNPs percent: G’—CMP-NP-50 (50% MNPs): 3300 ± 300 Pa, CMP-NP-70 (70% MNPs): 2000 ± 200 Pa; G”—CMP-NP-50: 90 ± 20 Pa, CMP-NP-70: 110 ± 20	Uva et al. [110]
	Carboxymethylcellulose (CMC) polymer and Fe_3_O_4_ MNPs functionalized with aminopropyl silane	Drug delivery	In vitro: MG-63 cell line	SQUID (Quantum Design MPMS): *M*_s_ 16 Am^2^/kg at 2.5 K and 14 Am^2^/kg at 300 K	The release of doxorubicin was investigated in the absence of a magnetic field, under the effect of an AMF (40 kHz, 2 mT) or a static field (SMF) (0.5 T). The best drug release kinetics was evidenced under the AMF condition	Rheological parameters: G’ = 3250 ± 120 Pa, G” = 258 ± 30 Pa	Uva et al. [111]
Shear-thinning	Hyaluronan (HA) filled with alumina (Al_2_O_3_) and multicore MNPs (MCPs). MCPs were characterized by clusters of superparamagnetic FeO_x_ nanoparticles	Possible application for MHT treatment and bioprinting	In vitro: mouse embryonic fibroblast cell line (ATCC CRL-1658 NIH/3T3, USA) for cytotoxicity investigation and bioprinting with BALB/3T3 mouse fibroblast cell line	-	Laboratory-made AMF generator system (signal generator Agilent 33512 (Keysight Technologies, Santa Rosa, CA, USA), RF broadband amplifier AR RF/Microwave Instrumentation 800A3A, induction coil (90 mm diameter), magnetic field sensor, interchangeable capacitors) was used. MHT experiments were performed at 525 kHz and a variable magnetic field of 5.4 ÷ 9.4 mT, or at 1050 kHz and induction amplitude of 5.4 ÷ 7.4 mT. Temperature was measured with the monitoring system ReFlex 4, Neoptix (Qualitrol, Quebec, Canada). A SAR value of 25 W/g at 1050 kHz and an amplitude of 7.4 mT was obtained	Rheological parameters: A distinct shear thinning behavior was evidenced with viscosity modifications in the range of 10^2^ Pa·s with a shear rate range of 10^2^ Hz. A storage modulus of about 300 Pa was measured	Vitkova et al. [112]
	Polyacrylamide (PAM) matrix with Fe_3_O_4_ MNPs bonded to the crosslinked PAM network based on hydrogen bonding	Possible application for MHT	-	VSM (Lakeshore 8604, Westerville, OH, USA). Measurements were performed at room temperature and at a maximum magnetic field strength of 8 kOe. *M*_s_ for 5 wt%, 10 wt%, and 15 wt% was 3.41 emu/g (3.41 Am^2^/kg), 5.15 emu/g (3.15 Am^2^/kg), and 9.05 emu/g (9.05 Am^2^/kg), respectively. All the MGs exhibited a reduced coercive field	The magnetic induction heating was made based on an AMF set-up (power supply, water-cooling system, induction heating system (copper tube resonant frequency: 76 kHz, DC voltage: 53.5 V, maximum current: 28 A), and a solenoid with 8 turns and a diameter of 50 mm. A temperature of 44.4 °C was achieved in 600 s	Rheological parameters: The storage modulus G’ increases directly proportionally with the MNPs percent. The viscosity decreases from 10^3^ Pa·s to 10^0^ Pa·s for a frequency variation between 10^−2^ to 10^2^ Hz shear rate	Xuan et al. [113]
	Hydroxypropyl methyl cellulose (HPMC) and Fe_3_O_4_ loaded with doxorubicin (DOX)	pH and magnetic dual-response hydrogel for synergistic chemo-magnetic hyperthermia tumor therapy	In vitro: human umbilical vein endothelial cells (HUVEC) for biosafety evaluation, In vivo: nude mouse 4T1 mouse breast cancer xenograft model—40 female SPF nude mice (weight 20 ± 0.3 g) injected with 0.1 mL of 4T1 cells	-	A custom-built MHT machine (frequency: 400 kHz, output power: 7.2 kW, coil diameter: 10 cm) and an infrared thermal imaging instrument Fortric225 (Fortric Technology, Santa Clara, CA, USA). A temperature higher than 41 °C was obtained for 10% MNPs concentration. To analyze the in vitro drug release, the physiological media PBS exhibiting different pH values (7.4 and 5.5) were chosen. AMF was also applied, and an increase in DOX release of about 57.6% was determined for the pH of 7.4 in PBS at 24 h, while for the pH of 5.5 the cumulative DOX release was estimated at 78.8% in the same conditions	Rheological parameters: The shear-thinning property of the MG proved that during the injection procedure, when the stress strengthened (0 s ÷ 1000 s) the hydrogel becomes thinner (30 ÷ 5 Pa·s shear viscosity). A good injectability of the MG was established	Zhou et al. [114]
	Montmorillonite colloidal gel with MNPs loaded with DOX	Postoperative treatment of hepatocellular carcinoma based on minimally invasive MHT	In vitro: NIH3T3 fibroblast cell line derived from mouse embryos In vivo: ICR HepG2 tumor-bearing mice (6 ÷ 8 weeks old), New Zealand rabbit with VX2 liver cancer	-	For MHT the rabbit animal model was exposed to AMF (312 kHz, 30 kA/m) for 15 min based on a heating equipment (Shuangping SPG, Shenzhen, China). The MG exhibited a *M*_s_ value of about 50 emu/g. The combined targeted therapy (MHT + chemotherapy) proved to be a very good solution for recurrence control	Rheological parameters: G’ decrease from 1400 Pa to 200 Pa at a temperature increase from 25 °C to 60 °C; the G” value was kept constant at about 100 Pa for the same temperature range	Chen et al. [115]
	Xanthan gum with a molecular weight of 2.5 × 10^6^ g/mol with superparamagnetic Fe_3_O_4_ MNPs and terbinafine (antifungal drug) incorporated	Thermal-induced control of drug release and *T*_2_-MRI contrast increase for possible treatment based on MHT and MRI imaging of skin carcinoma	In vitro cytocompatibility: human dermal fibroblast (HDF), human skin carcinoma (A431) cell lines. Antifungal performance: *Candida alibicans (C. albicans)* (ATCC 24433)	SQUID-VSM (Quantum Design, San Diego, CA, USA). Hysteresis cycles were measured at 300 K with a magnetic field variation ranging from −20 kOe to +20 kOe (1.6 MA/m). The temperature-dependent magnetization was measured under a magnetic field of 100 Oe in the 1.8 ÷ 300 K temperature range and zero-field-cooled (ZFC) and field-cooled (FC) conditions. *M*_s_ was about 2.6 emu/g (2.6 Am^2^/kg), remanence *M*_r_ of 0.12 emu/g (0.12 Am^2^/kg), coercivity *H*_c_ of 0.04 kOe (3.2 kA/m). ZFC and FC measurements confirm a superparamagnetic behavior with a blocking temperature (*T*_B_) of 98 K. MHT devices: DM1 calorimeter module from NanoScale Biomagnetics for AMF (890 kHz, 20 mT), temperature measurement sensor made of optical fiber for 15 min MRI measurements: MR Solutions 3.0 Tesla benchtop system	When the AMF (869 kHz, 20 mT) was applied, a SAR value of 100 W/g and an increase in temperature from 10 to 30 °C were determined. The release profile of terbinafine was analyzed under the action of an applied AMF (262.1 kHz, 23 mT) and AMF (174.5 kHz, 23 mT). Due to the local temperature increase around the MNPs under the AMF effect, it forces the relaxation capacity of the polymeric chains, leading to an improved diffusion of the drug. In addition, a drug release behavior depends on the applied field frequency. A higher release was associated with an increased frequency. Regarding the *T*_2_-MRI enhancement, it was noticed when the MNPs concentration was increased under the effect of a 3.0 T MRI scanner device	Rheological parameters: after drug incorporation G’ was higher than G” with an almost constant variation independent of frequency (rad/s). The same observation was maintained during the strain variation, which evidenced the solid (gel)-liquid (viscous fluid) transition at 100% strain. The MG viscosity decreased from 100,000 Pa·s (0.001 Hz) to about 1 Pa·s at 1000 Hz, proving a good shear-thinning character	Ribeiro et al. [116]
Self-healing	Chitosan and telechelic difunctional poly(ethylene glycol) (DF-PEG) loaded with Fe_3_O_4_ MNPs	Application in drug delivery and image enhancement in cervical cancer therapy	In vitro test: HeLa cervical cancer cels	-	-	Rheological parameters were analyzed to prove the self-healing capacity. The elastic response of MG was analyzed under a strain amplitude sweep test. The critical strain region of G’ and G” was determined at γ = 200% and 100%, respectively. G’ decreases above this critical strain zone, evidencing the collapse of the gel network. After that, the MG was subsequently subjected to a large amplitude oscillatory force (γ = 200%, f = 1.0 Hz), and the G’ value decreased from 400 to 300 Pa, indicating a loose network (tan δ = G’/G” = 0.5 ÷ 0.6). After decreasing the amplitude (γ= 1%, f =1.0 Hz), the G’ recovered quickly to the initial value, and the MG exhibits the original state (tan δ = 0.15 to 0.2). Based on an NdFeB magnet, it was demonstrated that under a static magnetic field, broken MG pieces can be glued together due to MNPs’ intrinsic magnetism, and after the field removal, an integral self-healed gel was obtained. High biocompatibility and cell viability were reported	Zhang et al. [128]
	Biopolymer containing xanthan gum (XG) crosslinked with chitosan (CS) with modified magnetic Fe_3_O_4_@SiO_2_	Possible application in cancer therapy	-	-	-	The self-healing property consisted of hydrogel property to withstand heavy counterweights (11.78 g), and after they were applied, a full shape recovery was achieved	Sanoh et al. [125]
	Alginate hydrogel platform loaded with Fe_3_O_4_ MNPs	Photothermal therapy for colorectal cancer	In vitro: CT26 murine colon cell carcinoma	-	The photothermal properties of MG (200 μg/mL Fe concentration) were investigated. Based on an 808 nm laser at various power densities (0.5, 1.0, and 1.5 W/cm^2^), MG irradiation was performed for 5 min. In addition, Fe concentration was set at 100, 200, 350, 500 μg/mL and irradiated with an 808 nm laser (1 W/cm^2^) for 5 min. The laser was turned on/off at every 5 min for 50 min. For the in vitro analysis, cells were seeded together with MG extracts for 12 h. Then they were irradiated with the NIR laser for 5 min. A good photothermal effect was obtained	As future investigation self-healing properties of the proposed MG will be further developed	Ji et al. [129]
Mechanical properties	Fe_3_O_4_@Fe-alginate/polyacrylamide (PAAm)	Possible guided catheter used in different magnetic navigation systems		VSM (735 VSM Controller, LakeShore, Westerville, OH, USA) with a maximum magnetic field strength of 9 kOe (716.2 kA/m) at room temperature. By modifying the MNPs percentage between 1 wt% and 20 wt%, the *M*_s_ varied from 2 emu/g (2 Am^2^/kg) to 17 emu/g (17 Am^2^/kg). A NdFeB permanent magnet was used to control the hydrogel movements	To investigate the MG behavior in external applied fields a sensor with a length of 100 mm and a diameter of 6 mm was moved by a NdFeB magnet. Under constant magnetic field influence, the MG navigated due to the strong magnetic attraction forces and low friction coefficient	Mechanical properties: for the MG with 1.0 and, respectively, 20.0 wt% MNPs were: 11.4 ± 1.5 rupture tensile strain and 0.915 ± 0.053 MPa tensile strength, 2.7 ± 0.4 and 0.201 ± 0.009 MPa, respectively. The MG can recover to its original shape without breaking after 90% compression with a compressive strength of 3.1 ± 0.2 − 5.6 ± 0.6 MPa and ~200 kPa compressive modulus from the same MNPs concentration. The fracture- energy decreases with increasing the MNPs percent 1550.5 ± 194.9 ÷ 2814.0 ± 69.6 Jm^2^	Haider et al. [62]
	Polyacrylamide with carbonyl iron	Secretions of proangionenic molecules and dynamic control of osteogenesis. Possible application in cancer therapy after tumor surgery to regenerate the operatory site	In vitro: Mesenchymal stem cells (MSCs)	-	Shear rheometry was involved in analyzing the MG’s mechanical properties under magnetic field action	Mechanical properties: 1 min. cycle testing condition: 30 s at 0 T and 30 s at 0.75 T. G′ at 0 T ranged between 0.1 and 0.14 kPa, and at 0.75 T reached 60 ÷ 90 kPa. The gel recovered its elastic modulus at 0 T with each cycle	Abdeen et al. [94]
	Poly(*N*-isopropylacrylamide) (PNIPAm) embedding Fe_3_O_4_ MNPs	Application in hyperthermia cancer therapy for melanoma	In vitro: A375 human malignant melanoma cells	Alternating gradient magnetometer (MicroMag TM-2900, Princeton Instruments), commercial induction heating system (Easyheat 224, Cheltenham Induction Heating), IR camera (Fortric 285#L21)	The temperature increases from 11.4 °C to 86.5 °C at 100 s, when the MNPs content in MG varied between 3 wt% ÷ 20 wt%. The saturated magnetization increases from 31 emu/g (31 Am^2^/kg) to 120 emu/g (120 Am^2^/kg), for the same concentration of MNPs. The MG can wrap the tumor during mechanical deformation, resulting in a uniform tumor heating process	The shape shifter structure can simultaneously encase and kill cancer cells (through magnetic hyperthermia. About 50% of cancer cells can be destroyed by applying MHT based on MG behavior under mechanical deformation. The link between mechanical deformation and MG shape under AMF action could exhibit an important discovery in cancer therapy	Tang et al. [119]
Porosity	Terephthaloyl thiourea crosslinked chitosan with Fe_3_O_4_ MNPs	Application dedicated to MHT	-	VSM analysis. *M*_s_ was reported to be about 66.92 emu/g (66.92 Am^2^/kg). The hyperthermia possible application was performed based on NATSYCO, Iran device	AMF with a frequency of 200 kHz applied for 1 mg/mL sample generated the highest SAR of about 78.43 W/g. The minimum SAR was achieved for a 10 mg/mL sample at a frequency of 350 kHz, corresponding to a value of approximately 7.51 W/g	A porous hydrogel was developed. Fe_3_O_4_ MNPs have covered the porous structure of MG. The localization and distribution of MNPs on the surface of MG within the channels are well observed based on scanning electron microscopy	Eivazzadeh-Keihan et al. [120]
	Silk fibroin loaded with doxorubicin hydrochloride (DOX) and loaded with Fe_3_O_4_ MNPs	The ability to release drug ability under different static or alternating magnetic fields for kidney cancer treatment in children	In vitro: Human fibroblast cells isolated from the foreskin by enzymatic digestion and Wilms’ tumor (nephroblastoma cells)	-	At about 35 days, the DOX release from MG was more than twice faster (47.33%) than in the absence of MNPs (21.12%). In addition, the DOX release increased in the presence of AMF	The average size of the pore and the overall porosity have an important influence in addition of MNPs. The total porosity was estimated at 23.59 ± 8.123% with an average pore size of about 188.299 μm^2^	Haghighattalab et al. [121]
	Macro porous alginate ferrogel microbeads	Drug release possible application for breast cancer treatment	In vitro: 4T1 cells (murine breast cancer cell line used for triple-negative breast cancer analyses)	-	A solid NdFeB magnet with a magnetic induction of 500 mT maintained for 1 min every 10 min showed a burst release of mitoxantrone	The manufacturing of macroporous hydrogels exhibiting an increased porosity and mechanical properties that are maintained during injection process is considered today an important challenge	Shin et al. [122]
Biodegradability/Biocompatibility	Poly(vinyl alcohol) (PVA)/water-soluble tricarboxy cellulose (CO)/Fe_3_O_4_ MNPs stabilized with a hydrophilic double layer of oleic acid (OA) molecules and dispersed in distilled water	Potential application for cancer therapy	-	VSM (ADE Technologies VSM880). Measurements were performed at room temperature with an applied field variation of +/−1000 kA/m. *M*_s_ was comprised between 0.0539 emu/g (0.0539 Am^2^/kg) and 0.9147 emu/g (0.9147 Am^2^/kg) as a function of MNPs quantity. Magnetic permeability determined at the saturation point μ_sat_ was between 0.345 memu (0.345 μA/m^2^) and 7.226 memu (7.226 μA/m^2^)	-	The biodegradability was evidenced through thermogravimetric analysis. Firstly, the first weight loss level was noticed at 50 ÷ 120 °C and corresponded to water evaporation. Then the second stage characterized by a major weight loss was observed between 220 ÷ 320 °C and corresponded to the structural degradation of PVA and CO/-	Baron et al. [126]
	Poly (L-lactic acid) (PLLA) microspheres combined with magnetostrictive cobalt ferrites (CoFe_2_O_4_) introduced in oil-water emulsion	Analysis of magnetoelectric/magneto-mechanical effect on cell development	In vitro: MC3T3-E1 cells	VSM (MicroSense EZ7, Lowell, MA, USA). Magnetic hysteresis loops and saturation magnetization were determined at room temperature for a field variation between +/−6000 Oe (477.4 kA/m). *M*_s_ was estimated at about 3 emu/g (3 Am^2^/kg)	Cell magnetic stimulation in a special bioreactor (1 Hz frequency and 1 mm amplitude for 16 h followed by non-active time of 8 h)	-/Biocompatibility was investigated by preparing cell culture under magnetic stimulation Magnetic stimulation was characterized by two effects: magnetostrictive variations of the MNPs propagated through the PLLA matrix and directly linked to surface charge variations through the magnetoelectric effect. In this way, it mimics the mechanical stress variations, which occur at the natural human body movements	Carvalho et al. [127]
	Iron (III)-alginate MG	Emission of Fe^3+^ ions under different ionic conditions and with important contribution in triggering the ferroptosis process in cancer therapy	-	VSM (LakeShore 8604, Westerville, OH, USA) with a measurement precision of 10 ÷ 7 emu. The maximum magnetic flux density was chosen to ±2 T. The maximum *M*_s_ was about 1.5 emu/g	After placing the MG into an acetic acid solution (pH = 3), the saturation magnetization decreased to 0.08 emu/g (0.08 Am^2^/kg)	It was concluded that after insertion in an acid, the superparamagnetic behavior is maintained, but the magnetization is greatly decreased in the absence of an applied magnetic field. This application is useful for the analysis of iron ion release with high impact on ROS generation	Chen et al. [130]

HCC—human/animal hepatocellular carcinoma cell line [131], L02—human fetal hepatocyte cell line [131], OVCAR3—huma epithelial cells from malignant ascites of a patient with ovary adenocarcinoma [131], NIH3T3—fibroblast cell line derived from mouse embryos [131], 3T3—immortalized mouse embryonic fibroblast cell line [131], HEPG2—epithelial-like human hepatocellular carcinoma cell line [131], A431—human epidermoid carcinoma [131], HeLa—human cervical cancer cell line [131], CT26—murine colon carcinoma cell line, A375—human malignant melanoma cell line [131], MG-63—human osteoblast-like cells [131].

## 4. Biomedical Applications of Magnetic Hydrogels in Oncology

**Figure 4:**
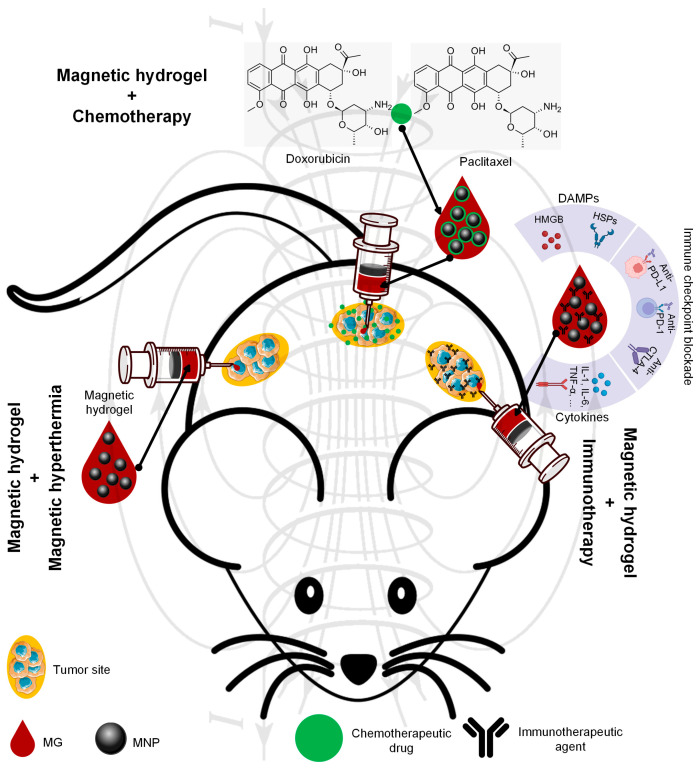
Biomedical applications of MG in oncology. This figure was generated using images assembled from Servier Medical Art, which are licensed under a Creative Commons Attribution 3.0 unported license (https://smart.servier.com, accessed on 31 August 2025) and from Xiong et al. Adapted from Ref. [19]. (Abbreviations: DAMPs—damage-associated molecular patterns: HMGB—high-mobility group protein, HSPs—heat shock protein; Immune checkpoint blockade: Anti-PD-L1—immunotherapy drug for blocking of programmed death-ligand 1 (PD-L1), Anti-PD-1—immunotherapy drug for blocking of programmed death-1 (PD-1), Anti—CTLA-4—immune checkpoint inhibitors for blocking the cytotoxic T-lymphocyte-associated protein 4 (antigen 4); Cytokines: IL-1—interleukin-1 activates lymphocytes and macrophages by promoting inflammation, IL—6—interleukin 6 acts as pro-inflammatory agent and regulates immune cell functions and metabolism, TNF—alpha—tumor necrosis factor alpha—pro-inflammatory cytokine).

In this review section, we will describe some oncological applications, such as magnetic hyperthermia, targeted drug release, and immunotherapy, or a combination of these approaches (Figure 4), and emphasize the importance of magnetic hydrogel properties with a focus on in vitro and in vivo investigations. Regarding the MG application in oncology, the field has learned that the best clinical performances are obtained when targeted drug release is combined with magnetic hyperthermia or immunotherapy, a structural control is directly linked to adequate mechanical properties, bio-mimicry of natural tissues and a spatiotemporal control must be followed and last but not least the fact that iron oxide are the most used MNPs due to their increased biocompatibility. Still controversial are the optimal concentration of MNPs, the temperature range adequate for biological media, and the long-term effects of treatments. To name a few “take-home” criteria, one should note that therapeutic temperature control is important; the physical laws governing optimal drug release patterns should be carefully analyzed across various viscosities; and long-term biocompatibility and biodegradability should be properly established.

### 4.1. Magnetic Hyperthermia

As an explicative term, “hyperthermia” consists of generating an increased temperature above 41 °C in the tumor zone with important cytotoxic effects on cancer cells [17,25,132]. When MNPs are incorporated into a hydrogel polymer matrix, they permit the assembly to produce, under an external AMF influence, a localized tumor heating phenomenon. Regarding the AMF parameters, although there is currently no standard limit for the maximum value of the product between magnetic field strength and frequency (*H* × *f*), it is recommended not to exceed the superior limit of 5 × 10^9^ A/(ms) in the case of biological applications [32,33,133]. It is well known that hyperthermia could have favorable effects on cancer detection and treatment due to its chemosensitizer and radiosensitizer attributes. These combined approaches proved over time to be efficient for cervical cancer, bladder cancer, breast cancer, melanoma, and head–neck cancers [25,134,135]. Usually, hyperthermia activates DNA repair pathways for healthy cells, followed by a good systemic immune response. On the other hand, in the neoplasm zone, tumor growth is diminished with a reduction in oxygen and nutrient flow capabilities in conjunction with a decrease in the formation of new blood vessels in the affected area. In clinical practice, one can identify different hyperthermia systems as a function of the frequency range, as follows: ultrasound, radiofrequency (RF), infrared (IR), and last but not least, microwave domain [134].

As already described by V. Manescu (Paltanea) et al. [25] and H. Gavilan et al. [26], the hyperthermia mechanisms are based on eddy currents, which occur inside the electrically conducting samples, such as the human body, and follow circular patterns, magnetization vector reversal for MNPs, and dipolar motions of magnetic dipoles. For the MG particular case, because polymer macrochains are physiosorbed on the MNPs surface, a restriction regarding rotation and movement occurs, hindering the Brownian relaxation process for dipole movement. So, as a direct consequence of the physical theory, the main mechanisms that are responsible for the heat induction are the eddy currents and the so-called Neel relaxation processes [25,26]. Tang et al. [73] manufactured a magnetic double network hydrogel characterized by good mechanical properties and excellent ion resistance dedicated to drug delivery and MHT treatment. They proved that no Brownian effects occurred since these are characterized by simultaneous rotation of MNPs. Regarding the Neel relaxation phenomenon that includes only spin magnetic moment rotation, by maintaining the MNP fixed due to gel chain restriction, a heating effect was obvious. In addition, the developed MG proved to be applicable for the MHT approach, generating a temperature increase of 15 °C in only 3 min. The authors [73] concluded that the hyperthermia effect was initiated based on induced eddy currents and the Neel relaxation mechanism.

The SAR, defined as the heat quantity generated by a sample during a certain period of time, represents an important parameter in MHT and a measure of the treatment efficiency (Table 3). Usually, there are searched magnetic hydrogels that exhibit high SAR values. The most important factors that influence SAR are the relative magnetic permeability, size, and shape of the MNPs and the frequency of the AMF. Meenach et al. [136] made a magnetic hydrogel with a PEG matrix that exhibited a high temperature increase (>61 °C) considered beyond classical MHT (>41 °C) with thermoablative effects that generated M059K glioblastoma cell death. The same research group has analyzed the controlled synergistic delivery of paclitaxel chemotherapeutic drug in combination with MHT generated under a magnetic field effect in poly(β-amino ester)/iron oxide MG [137]. An AMF (294 kHz, 17.4 kA/m) was applied for 5 min, and an increase in local temperature with a maximum value of 55 °C was achieved. It was noticed that the temperature range could be adjusted by controlling the magnetic field parameters, such as maximum magnetic field strength and frequency. Regarding the paclitaxel release, a near-zero-order release model type was adopted, and it was concluded that it can be controlled as a function of MG degradation because after 60 ÷ 70% release of the total quantity, the drug delivery slowed. Other studies providing SAR values for MHT applications are given in Table 2.

For the MG case, an important problem that must be addressed consists of MNPs’ agglomeration, which could be challenging in some cases. Among the factors that exhibit an influence on MHT based on MG is the crosslinking density. In this direction, the study of Eivazzadeh-Keihan et al. [138] showed that changing the crosslinking density in chitosan hydrogel combined with MNPs led to different nanostructural features and SAR values. A higher crosslinking density influenced the crystalline phase, MNPs nanostructure, and magnetite content when the gel was introduced in FeCl_2_ solution. A reduced Fe^2+^ uptake and nucleation sites for Fe_3_O_4_ were noticed, together with the fact that at alkaline pH, Fe^3+^ ions were adsorbed on chitosan functional groups. This study evidenced that an increased crosslinking density could lead to a reduced quantity of MNPs and a decreased SAR value [138].

Another important key feature is the hydrogel sol–gel transition with high impact on delivering molecular payloads as well as non-invasive implantation in different body parts. Injectable MGs are preferred because they are injected as a solution, and then a gel transition takes place at the human body temperature. Le Renard et al. [139] manufactured an injectable MG made of chitosan and a block copolymer (poloxamer 407). It is well known that block copolymers exhibit a good sol–gel transition (33 ÷ 36 °C) with a temperature modification (37 °C) after injection because of the hydrophobic segment interactions. The authors [139] introduced 20% (*w*/*v*) superparamagnetic nanoparticles (SPIONs) and then injected the MG locally at the tumor site. In vivo studies were performed for female CB17 SCID mice and female Swiss nude mice. The tumor xenograft was inoculated in CB17 SCID mice. It was based on human invasive breast ductal carcinoma with negative steroid receptor status (SK-BR-3). In contrast, for the Swiss mice group, SKOV-3 and LN229 cell lines led to dense ovarian carcinoma and glioblastoma tumor appearance. Because the amount of MNPs was considered too low, the sol–gel transformation was compromised, resulting in reduced mechanical strength and instability. On the other hand, when the MNPs quantity was increased to 40% (*w*/*v*), it was estimated to be a favorable outcome for MHT application. It can be concluded that MNPs act as reinforcement points, which maintain the mechanical stability of the hydrogel and add supplementary magnetic properties, permitting different optional features, such as heating or controlling under AMF influence, to occur.

Strongly linked to the MG injectability is also the shear-thinning property because hydrogels are usually pressed through a small-diameter needle to be inserted into the body and are subjected to high shear stress. Based on shear thinning and the fact that MNPs significantly influence the MG rheological behavior by imposing different viscosities, various cases should be analyzed to establish an optimal MNP diameter and concentration as presented above. The study of Qian et al. [105], which manufactured an injectable MG based on silk fibroin blended with iron oxide MNPs, proved that because of the shear-thinning property, the MG was very easily injected directly into the tumor area, proving to be very adequate for MHT treatments. In addition, it was noticed that the hydrogel caused the blood vessels that fed the tumor to close, thereby cutting off the blood supply in the vicinity of the neoplasm.

Table 3 presents some examples of the MHT approach used at the pre-clinical level based on MGs. One can immediately notice that most of the medical approaches combined MHT with chemotherapy because drug release seems to be enhanced when an external AMF is applied [63,114,140,141,142,143,144,145,146,147]. Another aspect that is worth mentioning consists of the fact that doxorubicin is the most used chemotherapeutic drug, and many studies are performed on the topic of breast cancer. There are involved cell lines such as MCF-7, BT549, and MDAMB, which are characterized by hormone-positive receptors and protein HER2-positive, or triple negative character, respectively. The control cell lines are usually of soft tissue murine origin with a decreased growth speed compared to human cancer cell lines. These types of investigations proved that MHT could be a useful and effective approach in most types of breast cancer with various AMF conditions and different SAR values, as presented in Table 3 [63,114,140,141,142]. In addition, we found some MHT applications for hepatocellular carcinoma, which are based on in vivo studies made on mouse and rabbit animal models with xenografted tumors derived from human cell lines. The main finding of these studies [115,144,148] was that the tumor growth was inhibited even when the MHT was used as a solo therapy, leading to cancer cell apoptosis. These types of medical strategies could also be applied in colon or ovarian cancer treatment and head and neck neoplasms, as stated in [25,143].

By analyzing some practical implementations of MHT treatments, it can be concluded that a rigorous estimation of their outcome regarding the biological impact is of utmost importance. A key challenge consists of overcoming the so-called heat shock proteins (HSPs) that are defined as endogenous proteins with a significant role in tumor cell stabilization under external stress and by protecting the neoplasm tissue from the thermal solicitations produced through MHT application [149].

### 4.2. Drug Delivery

Hydrogels are characterized by some unique attributes due to their porosity, making them prone to drug inclusion and controlled release under specific environmental conditions [150,151]. The release of chemotherapeutics is a function of hydrogel diffusion laws [152,153]. Initially, the drug molecules are incorporated into the hydrogel matrix when it is submerged in a drug solution [154]. Under certain external stimuli such as solution pH, temperature, electric field, light, different enzymes, magnetic field, or saline environment, the hydrogel porosity and internal structure can be handled [155,156,157]. After that, the drug molecules are released from the hydrogel matrix according to Fick’s diffusion law or other anomalous diffusion kinetics [158].

In particular, magnetically responsive hydrogels are a new and interesting tool in drug release, which addresses some of the limitations met in the case of classical hydrogels. One can notice that MGs are suitable for a much more targeted and controlled medicine release. In this way, a localized procedure with low side effects on healthy tissue is foreseen. By including MNPs into the hydrogel matrix, a magnetothermal effect under AMF influence could occur as presented before. In addition, an accelerated drug diffusion is possible due to MNP dipole orientation, which generates deformation effects and mechanical vibrations, inducing a local stress that enhances the drug release [159].

Multi-stimuli responsive MGs are an adequate approach to the drug theragnostic domain, especially in cancer treatment, by integrating the different stimuli mentioned before in a synergistic process that influences the tumor microenvironment via thermal variations, acidic pH existence, and redox conditions. By including MNPs into the hydrogel structure, it is possible to guide the MG directly to the tumor under a magnetic field influence and control the drug release only into the cancerous cells. Usually, many of the applications consider pH-responsive behavior. At a physiological pH of 7.4, the MG is stable with low drug release. In contrast, when the pH decreases in the acidic zone, the MG matrix begins to swell or degrade, resulting in an accelerated drug release in the tumor environment. Some studies [63,114,140,141,142,143,144] presented in Table 3 clearly demonstrated this type of combined behavior under different pH values. An interesting study was presented by Huang et al. [160], who developed a triple-responsive MG to pH, glucose, and temperature. A self-regulatory drug release with a conservative approach was observed.

Other approaches involve the redox-sensitive components of MG, which are influenced by high levels of reducing agents such as glutathione (GSH). This contributes to the polymer chain break, leading to MG disintegration and accelerating drug release, with minimal impact on healthy tissue in the tumor vicinity. Dong et al. [161] prepared a hydrophilic core–shell magnetic micro-organogel with thermo/GSH–sensitive response dedicated to hydrophobic drug release. It was concluded that the main mechanisms that governed the targeted chemotherapy were a combination of degradation, erosion, and diffusion of the polymer.

Usually, in the literature, there are two approaches found, which establish a link between the magnetic field application and drug release. Firstly, under the action of an external uniform magnetic field, the MNPs form a needle-like structure in the hydrogel with a parallel or perpendicular alignment with respect to the drug diffusion pathway. The parallel direction favors the highest drug release, since the lowest drug dose characterizes the perpendicular direction. A representative study was conducted by Liu et al. [159], in which MNPs and gelatin were combined for B12 vitamin release. The authors [159] investigated different alignment directions, such as perpendicular, parallel (anisotropic magnetic behavior), and random (magnetic isotropic behavior). When the nanochannels of the MG were oriented parallel to the drug permeation, an important increase (2.2 times) in the permeability value (480 × 10^6^ cm^2^/min) compared to the case of perpendicular orientation (223 × 10^6^ cm^2^/min) was achieved. In addition, when compared to the random magnetic particle’s orientation (306 × 10^6^ cm^2^/min), a 1.6 times increase was determined. It was concluded that controlling the nanochannel formation in MG by applying a magnetic field is an efficient tool to obtain strict control of drug release. Secondly, another approach consists of toggling the MG activation for the control of drug release by alteration of the applied magnetic field parameters. Due to the fact that hydrogels exhibit two states, sol and gel, an “on-off” mechanism could be considered. In Ref. [76], porous alginate ferrogel microbeads enhanced with MNPs proved to be able to act as a switch in the drug release process. The existence of covalent crosslinking determined good mechanical properties during injection administration, while the macropores generated an increased loading with biomolecules under magnetic field action.

The swelling property could be modified by choosing different crosslinking densities or various polymer compositions. Hamidian and Tavakoli [162] manufactured a cellulose-based MG with properties such as swelling and deswelling used to control the 5-Fluorouracil (5-FU) chemotherapeutic drug. At the application of an external magnetic field, the MNPs inside the MG were attracted and formed agglomerations, resulting in a significant reduction of drug release due to a decrease in the total volume and pore sizes. On the other hand, when the magnetic field was switched off, the drug release rate increased to a high amount. It was concluded that this type of MG with controlled swelling property under magnetic field influence represents a useful tool in chemotherapy [163].

As presented in the previous section, the drug release property was combined with MHT. In this direction, Wu et al. [103] developed an MG manufactured by integrating alpha cyclodextrin (α-CD) onto the copolymer section PEGylated magnetite MNPs based on a “sol-gel” transition that enhanced the MHT property of Fe_3_O_4_ particles. The authors [103] incorporated a hydrophilic drug, doxorubicin (DOX), and a hydrophobic one, paclitaxel (PTX), and analyzed the drugs’ release profile and efficiency on a 4T1 breast cancer mouse model for 15 days. No recurrence of cancer occurred even 60 days before treatment, evidencing a very good effect of drug release in the presence of magnetic hyperthermia.

In Table 3, examples extracted from the literature on drug delivery are provided [76,103,121,164,165,166,167,168,169,170,171]. In one study [164], it was found that gene therapy associated with drug delivery and near-infrared (NIR) hyperthermia led to a high temperature of 44 °C being reached in about 5 min after NIR irradiation, with a high amount of tumor inhibition after only 2 cycles of hyperthermia. Based on in vitro investigations, the authors [164] concluded that different release rates of folic acid were due to hydrophobic or electronic interactions that bind siRNA and Au-coated iron oxide MNPs. Radio frequency AMF (300 kHz, 7 kW, 5 kA/m) proved to be very efficient in reducing the MDAMB231 cell viability (38%) by increasing the DOX release for only 15 min irradiation [165]. Different studies [168,170,171] developed magnetic hydrogels with a higher degradation rate at acidic pH conditions. These MGs were loaded with chemotherapeutic drugs and proved very efficient in the treatment of cervical, lung, and head and neck cancers. The low-frequency alternating magnetic field (LAMF) influence on DOX release was analyzed using the MG-63 cell line. In the absence of LAMF, only about 40% DOX release was measured, while at the LAMF application, an increased DOX release of 67.2% was observed with a high impact on cell viability decrease. Kim et al. [169] chose to investigate the LAMF effect on the SCC7 cell line and found that the cumulative DOX release was about 60% within 120 min. Regarding in vivo investigation, a significant tumor reduction was achieved under LAMF stimulation.

One can conclude that the development of multi-stimuli magnetic hydrogels could be applicable in different drug targeting strategies with important implications on tumor growth reduction and providing new platforms for drug release.

### 4.3. Immunotherapy

Immunotherapy is considered today one of the newest and most promising tools for cancer treatment. It generates immune activation, which fights against tumor growth and secondary cancer apparition. Some of the most used strategies are the use of cancer vaccines and checkpoint inhibitors. The first method mentioned consists of educating or enhancing the immune cells that can attack the cancer tumor, while the second strategy corrects the dysfunctional immune pathways that are present in the tumor medium [172]. In comparison with the traditional cancer approaches, immunotherapy is characterized by reduced toxicity and increased efficiency [173,174], but usually these types of drugs are used as second-line treatments. However, the solution of checkpoint inhibitors is expected to be adopted as a first-line treatment, despite drawbacks such as high cost and different responses from the patient’s body, limited during its application on a large scale [175,176,177,178]. The most important challenges met in immunotherapy are the drug’s inability to enter solid tumors and characteristic immunosuppressive tumor microenvironments that are directly linked to reduced immune activation. In these cases, a correct administration dose should be carefully checked [179,180]. The involvement of a magnetic system in immunotherapy treatments exhibits improved spatiotemporal control regarding immunomodulatory drug transport, release, and concentration with diminished side effects [181].

Hydrogels can be used to sustain a continuous effect through in situ tumor bed implantation to determine a long-lasting immune response. There are just a few studies in the literature that analyze magnetic hydrogel efficiency in a direct relationship with immunotherapy. Wang et al. [182] developed a magnetocaloric-responsive hydrogel used for pyroptosis-relay immunotherapy to reduce the apparition of metastases and recurrence. They manufactured a gelatinic–tannic acid-based gel crosslinked with a bimetallic nanoalloy (Zn_0.35_Fe_0.65_) Fe_2_O_4_, which should be locally applied after surgery. Under the influence of AMF, the MHT phenomenon occurred, and the emission of Fe^3+^, Fe^2+^, and Zn^2+^ ions induced a strong pyroptosis effect. After that, the intracellular content, cytokines (IL-18, IL-1β), and debris generated by pyroptosis were released to the lymph nodes and triggered immunogenic cell death. By combining with PD-1 checkpoint blockade, a reduction in tumor recurrence and metastasis was achieved by analyzing a preclinical model of B16F10 tumor-bearing mice. This animal model exhibited a long-lasting immunological response. Regarding the in vitro studies, melanoma skin mouse tissue cells (B16F10) in the presence of high-concentration MG extracts and in the absence of an external AMF (500 kHz, 6.6 kW) were characterized by about 80% viability, while at the AMF application, the B16F10 viability decreased to 60%. In vivo investigations were performed on different mouse groups, and the highest tumor growth inhibition (90%) was achieved for the group injected with MG in combination with programmed cell death protein (PD-1) under AMF influence and with a median survival rate of 45 days. The authors [182] concluded that the developed MG + PD-1 could be successfully applied as adjuvant immunotherapy in melanoma treatment. In a recent study, Quan et al. [183] manufactured an immunomodulatory hydrogel that can induce a re-programming effect on tumor-associated macrophages to trigger immunogenic cell death of melanoma skin cancer. The authors [183] used the hyaluronic acid natural polymer in conjunction with borosilicate glasses (BGs) and Fe_3_O_4_ nanoparticles to neutralize the pH value and generate a pro-inflammatory response (M1) of macrophages to fight against cancer cells. This process is based on the fact that BGs are able to change the pH medium through alkaline group formation, such as SiO_4_^4-^ and BO_3_^3-^. A depletion of intracellular H^+^ and a remodeling of the immunosuppressive tumor environment followed. In addition, Fe_3_O_4_ determined the apparition of OH^-^ through Fenton-like reaction and enhanced the immunogenic cell death. The in vitro cytotoxicity was analyzed on L929 and B16F10 cells and extracts made with different BGs (5 ÷ 30 mg/mL) and Fe_3_O_4_ (50 ÷ 250 μg/mL) concentrations. It was noticed that at the maximum concentration of MNPs, a very low cell viability of less than 10% occurred, indicating a very good antitumor efficiency of the developed MG. The tumor recurrence possibility was analyzed based on in vivo investigations performed on subcutaneous tumor B16F10 induced in murine animal models. Based on chemiluminescence microscopy and histopathological analysis, a high tumor volume reduction with severe necrosis and cell apoptosis was found. It was concluded that the developed MG exhibited a good immunomodulator effect even in the absence of monoclonal antibodies and external AMF, which makes this product a non-expensive and easy-to-use one.

Another interesting analysis performed by Yan et al. [184] described the manufacture of a novel injectable Fe_3_O_4_@Dimercaptosuccinic acid (DMSA)@Platinum (Pt)@poly(lactic-*co*-glycolic) PLGA-PEG-PLGA magnetic hydrogel system that can be applied for magnetothermal therapy (MHT), chemodynamic therapy (CDT), and immunomodulation. This hydrogel exhibited a sol–gel transition at 37 °C. Under AMF (370 kHz, 30A) irradiation, a temperature of 50 °C was achieved in about 7 min and induced MHT-related cell death. In addition, by activating pH/temperature-dual responsive peroxidase (POD)-like activity in MNPs (Fe_3_O_4_@DMSA@Pt), the Pt chemodynamic activity led to an increase in the number of reactive oxidative species (ROS), and then a phenomenon of immunogenic cell death (ICD) occurred. ICD is directly linked to the sequential release of damage-associated molecular patterns (DAMPs), which contain calreticulin (CRT), high-mobility group box 1 protein (HMGB1), and adenosine triphosphate (ATP). The efficiency of these combined mechanisms was studied on the A20 cell line. It was concluded that this multi-purpose MG exhibited good results in inducing cellular death via different mechanisms and proved to be an efficient tool for diffuse large B-cell lymphoma treatment.

Massana Roquero et al. [185] made a localized “smart” delivery procedure of monoclonal antibody Trastuzumab (TmAb) based on two magneto-activated materials. The first one, manufactured of polyvinyl alcohol (PVA)-diboronate (DB)-interpenetrated (IPN) alginate (Alg) microgel nanocomposite (PVA-DB-IPN-Alg) loaded with magnetic nanoparticles (Fe_3_O_4_), behaves as a drug-delivery element, while the second one consisted of MNPs functionalized with the glucose oxidase (GOx) enzyme. When a static magnetic field was missing, the H_2_O_2_ quantity was decomposed by the catalase enzyme and did not interact with the alginate MG. Upon aggregation of these two types of particles induced by a permanent magnet, the GOx-MNPs produced H_2_O_2_ and degraded the PVA-DB-IPN gel. As a direct consequence, the pores of MG opened and released the monoclonal antibody. The magnetic actuation procedure was considered very efficient in breast cancer HER2+ immunotherapy treatment. However, the authors [185] concluded that supplementary in vivo investigations are necessary. They proposed improvements such as silica-based coatings for MNPs and adjustments of the distance between the external static magnetic field and MNPs inside the MG, when an animal or human body is involved.

Table 3 presents details extracted from the above-mentioned studies with a focus on in vitro and in vivo analyses [182,183,184]. It is worth mentioning that these investigations are dedicated to skin, blood, and breast cancer. One study [182] combined immunotherapy with MHT and used the immune checkpoint inhibitor PD-1, which represents a “chemical break” that influences the T cells to attack healthy body tissue, for melanoma treatment, while another study [183] investigated only the MG influence on the host immune system in the absence of chemotherapeutics and an alternating magnetic field. This last-mentioned investigation proved that only the innovative hydrogel components were enough to induce the melanoma cell apoptosis, making this type of approach less toxic and expensive when compared to classical treatments. In Ref [184], three cancer treatment strategies were combined and demonstrated to be an interesting treatment with fast positive outcomes.

In summary, different types of immunotherapies could be successfully combined with magnetic field action, but there is a lack of information in the literature. It is advisable to develop more of these types of studies due to their limited side effects and localized physical mechanisms, and try to apply them in clinical investigations.

**Table 3:** Oncological applications of magnetic hydrogels in pre-clinical practice.

Cancer Type	Application	Hydrogel/Chemotherapeutic Drug	MNPs/NPs	In Vitro	In Vivo	Properties	Clinical Relevance	Ref.
Breast cancer	MHT + Chemotherapy	Chitosan/Doxorubicin (DOX)	Ferromagnetic vortex domain iron oxide (FVIOs) (0.6 mg/mL)	L929 and MCF-7 (ER+, HER2+, EGFR+) cell lines	Mouse animal model	In vitro: 10 min. exposure to AMF (495 kHz, 220 Oe (17.507 kA/m)), SAR values 371 (150 Oe (11.936 kA/m)) and 856 (220 Oe (17.507 kA/m)) In vivo: DOX release under AMF effect: The absence of high step release of the drug proved the negligible influence of the AMF irradiation	*H* × *f* = 8 × 10^9^ A/(ms)	Gao et al. [63]
		Two gelatin-based pH- and thermal-responsive (PDMAEMA) or PDMAEMA/PNIPAAm hydrogels/DOX	Fe_3_O_4_	NIH3T3 and MCF-7 cell lines	-	In vitro: At an acidic pH of 4.2, both MGs exhibited a property of “smart” chemotherapeutic effect. When combined with MHT in an acidic medium, an increased drug release was noticed at 42 °C	pH-responsive (4.2) meets the condition of drug delivery at an acidic pH	Derakhshankhah et al. [140]
		Contractible HPMC/DOX	Fe_3_O_4_	HUVEC cell line	Nude mouse 4T1 mouse breast xenograft model	In vitro: AMF (400 kHz, 7.2 kW, coil diameter 10 cm) for 120 s determined an increase in temperature from 25 °C (0% MNPs) to 100 °C (40% MNPs) In vivo: temperature on the surface of the tumor center placed above the injection reached 48 ± 1.2 °C in 1 min. Then MHT expanded to the whole tumor surface, with a temperature value of 68 °C. The AMF enhanced the DOX release with no recurrence of cancer	pH-responsive (5.5) meets the existing recommendations	Zhou et al. [114]
		Poly (N-vinylcaprolactam) (PNVCL)/DOX	Iron oxide MNPs	L929, NIH3T3 mouse embryonic fibroblasts cell lines and MCF-7, MDAMB (ER-, PR-, HER2-) human breast cancer cell lines	-	In vitro: Radio frequency (RF) generator (250 kHz, 450 A) powered a coil of 6 cm diameter for 10 min. The MHT temperature of 43 °C was reached in 600 s in combination with a SAR value of 204 W/g. The cell death occurred in the presence of magnetic nanogel in a percent of 83% (MCF7), 87% (MDAMB232), 62%, and 68% for the healthy fibroblast cell lines, respectively. Under AMF action, the DOX release increased due to the synergistic effect of magnetic field oscillations that disrupted the cell membrane and permitted the DOX release	*H* × *f* = 0.11 × 10^9^ A/s per meter unit, pH-responsive (4.5) meets the existing recommendations	Indulekha et al. [142]
		β-cyclodextrin-graft-poly(Nisopropylacrylamide) (β-CD-g-PNIPAAm)/DOX	Fe_3_O_4_	MCF7	-	In vitro: *M*_s_ of MG was about 8.2 emu/g (8.2 Am^2^/kg). In physiological conditions, it was observed that a low drug release value due to strong hydrogen bonds between the drug molecules and the hydrogel network. By increasing the temperature up to 40 ◦C, the release amount was increased due to collapse of PNIPAAm chains. On the other hand, the release of DOX was slow, and within 24 h, only 33.8% of the total DOX quantity was released	The release rate kinetic meets the existing recommendations	Eskandani et al. [166]
		Poly N-isopropylacrylamide (PNIPAAm) nanosized MG/DOX	Oleic acid and oleyl amine-coated Fe_3_O_4_ MNPs	MDAMB231 cell line	-	In vitro: RF (300 kHz, 7 kW, 5 kA/m) absence: 50% cell viability was found when DOX was used, compared to 90% viability in the DOX absence RF applied for 15 min: cell viability was decreased up to 38%	*H* × *f* = 1.5 × 10^9^ A/(ms) meets the existing recommendations	Nandwana et al. [165]
		Magnetic supramolecular hydrogel from gellan gum self-assembled by PEGylated MNPs and α—cyclodextrin (α-CDs)/DOX + Paclitaxel (PTX)	PEGylated Fe_3_O_4_	RAW264.4, 4T1 (ER-, PR-, HER2-) cell lines	Female BALB/c mice (4 weeks, 20–25 g) with 4T1 xenografted tumor (2 × 10^6^, in 150 μL serum-free RPMI-1640 medium)	In vitro: the release rate of PTX was low at 2% per day over 15 days, and the MGs released sustainedly only ~30% of PTX for half a month. The release percentage of DOX from the hydrogel was about 60% in the first 2 days. The release was slow after that, and the total release percentage was more than 90% in a month. In AMF (400 kHz, 1.8 kA/m): DOX and PTX release varied between 49 ÷ 63% and 3 ÷ 13% in the first day, respectively; MHT (AMF applied for 80 s), a SAR value of 1334 W/g Fe was achieved In vivo: the cancer cells were constantly killed, and the inflammatory reaction disappeared after 4 weeks. No recurrence was noticed in the main organs of the animals. MG released steadily DOX and PTX for about 2 weeks after implantation. Survival rate at 20 days post-surgery was about 85%	*H* × *f* = 0.73 × 10^9^ A/(ms) meets the existing recommendations	Wu et al. [103]
	MHT + Controlled Drug Delivery (Methotrexate (MTX))	Poly (NIPAM-*co*-DEAEMA)/Anti-metabolite drug Methotrexate (MTX)	Bare surface Fe_3_O_4_ and TMSPMA surface modification Fe_3_O_4_	MCF-7 cell line	-	In vitro: AMF (375 kHz, 4 kA/m) exposure led to 42 °C in 220 s. A SAR value of 83.7 W/g was achieved. Up to 40 wt % of MTX was released at pH 5.5 and 37 °C	*H* × *f* = 1.5 × 10^9^ A/(ms), pH-responsive (5.5) meets the existing recommendations	Najafipour et al. [141]
	MHT	Nanobiocomposite hydrogel (chitosan, silk fibroin, polyvinyl alcohol)	Fe_3_O_4_	BT549 cell line (Erβ2+, Y5R+, TRPC4+, AR+), HEK293T cell line	-	In vitro: Oscillating magnetic field (100 kHz, 200 kHz, 300 kHz, 400 kHz). SAR values: 21 W/g (100 kHz), 35.72 W/g, 44.65 W/g, and 53.16 W/g (400 kHz)	The maximum SAR value at 400 kHz meets the existing recommendations	Eivazzadeh-Keihan et al. [138]
	NIR Hyperthermia + Gene therapy + Drug targeting (Folic acid)	Folate/polyethylenimine-conjugated poly(organophosphazene) polymer, which encapsulates small interfering RNA (siRNA)	Au-Fe_3_O_4_	MDAMB231 cell line	Female BALB/c nude mice (5 weeks old) with xenografted tumor (1 × 10^7^ MDA-MB231 cells)	In vitro: An increased weight loss was noticed within 7 days, and 32% of the hydrogel weight remained at 18 days after injection. The complete dissolution of the hydrogels occurred at 30 days post-injection. The different release rates could be linked to electronic and hydrophobic interactions that bind siRNA and Au-Fe_3_O_4_ MNPs In vivo: NIR irradiations (laser power 1.2 W/cm^2^) were applied in 2 cycles performed at one- and two-weeks post-surgery, and an increase in temperature of about 44 °C in about 5 min was achieved. The best tumor inhibition was reached in the case of two cycles of hyperthermia, which facilitates the release of drugs	Laser power meets the existing recommendations	Zhang et al. [164]
Colon cancer	MHT + Chemotherapy	Medical chitosan/DOX@Ferumoxytol (FMT)	Ferumoxytol commercial MNPs	HT-29 cell line	Male BALB/c nude mice (18 ÷ 27 g weight, 6 weeks old) with a colon xenograft tumor	In vitro: a temperature’s increase up to 42 °C was achieved. In PBS solution (pH of 5.2), a much slower DOX release was noticed at 43 °C compared to the MG behavior at 37 °C In vivo: MHT temperature of about 42 ÷ 43 °C was achieved after 20 min starting from 34 °C (380 kHz, 15 A). A good heating property was noticed only in the tumor region, with no harmful effects on healthy tissue	*H* × *f* = 0.05 × 10^9^ A/s per meter unit	Chen et al. [143]
	NIR + magnet+ enzyme response + Chemotherapy	Thermosensitive poly(N-acryloyl glycinamide) (PNAGA)/DOX and polyester (PE) capped mesoporous silica nanocarriers (MSNs) (PNAGA-DMP-Fe_3_O_4_@GO)	Fe_3_O_4_ nanoparticles grafted graphene oxide (GO)	LoVo cell line	Tumor-bearing nude mice	In vitro: the magnetothermal efficiency of MG was maintained even after five cycles of turning on and off the magnetic field. A temperature of 55 °C was achieved. NIR light permitted the right manipulation of nanocarriers’ release by changing the laser power. The esterase’s use triggered DOX release over 60% after 24 h incubation in the esterase group In vivo: the mice treated by PNAGA-DMP-Fe_3_O_4_@GO MG with NIR irradiation showed the best tumor inhibition with complete remission after 3 weeks	pH-responsive (5) meets the existing recommendations	Yuan et al. [167]
Hepatocellular carcinoma (liver cancer)	MHT + Chemotherapy	Gelatin/DOX	Magnetic zeolite-based imidazolyl skeleton (Fe@ZIF-8)	-	Female BALB/c mice (6 ÷ 8 weeks) with H22 tumor cells subcutaneously injected	In vitro: under AMF (312 kHz, 25 kA/m) influence, the temperature of MG with various Fe@ZIF-8 increased by approximately 24.1, 34.9, and 46.6 °C after 5 min of field irradiation. The DOX@Fe@ZIF-8 was characterized by magnetic heating- accelerated drug release. The ratio of released DOX increased with AMF exposure, up to 42.1% and 33.4%, under pH 6.5 and pH 7.4 In vivo: the temperature at the tumor site reached 42 °C within 10 min of AMF irradiation and gradually degraded in the tumor environment, concomitantly releasing DOX	*H* × *f* = 7.8 × 10^9^ A/(ms)	Du et al. [148]
		Gelatin/DOX	Clinically available montmorillonites and amphoteric gelatin nanoparticles	NIH3T3 cell line	VX2 tumor rabbits	In vitro: *M*_s_ of MG loaded with DOX was 55 emu/g (55 Am^2^/kg). The temperature increased to about 53 °C in 10 min. MG loaded with DOX released a high amount of drug (maximum value of 40%) under AMF, which was considered a double release of drug in the absence of AMF (maximum value of 18%) In vivo: after MHT application for 15 min., tumor tissue was collected and exhibited an important cell apoptosis	AMF (312 kHz, 30 kA/m) applied for 10 min *H* × *f* = 9.3 × 10^9^ A/(ms)	Chen et al. [115]
	MHT	Triblock copolymers of poly-((N-isopropylacrylamide-co-dopamine)-b-poly(ethylene-glycol)-b-poly(N-isopropylacrylamide-co-dopamine)) (Poly-(NIPAM-co-DOPA)-PEG-poly(NIPAM-co-DOPA), NDP)	Fe_3_O_4_	-	Subcutaneous liver cancer recurrence mouse model	In vivo: investigations under AMF (312 kHz, 25 kA/m) influence. The temperature of tumor sites rose from 30 to 46 °C within 15 min. This MHT result was considered enough and inhibited tumor growth. In the case of surgically induced recurrence, a reduction to 20% in 14 days was observed under AMF action	*H* × *f* = 7.8 × 10^9^ A/(ms)	Yan et al. [144]
Cervical cancer	Targeted drug release chemotherapy	Trangacanth gum (TG), poly(acrylic acid) (PAA) pH-responsive/DOX	Fe_3_O_4_ (MH1: 53.26% C, 42.28% O, 4.46% Fe; MH2: 48.57% C, 44.65% O, 6.78% Fe)	HeLa cell line	-	In vitro: negligible drug release (22.3% -MH2 and 19.7%–MH1) in normal physiological conditions (pH 7.4, T 37 °C); in cancerous conditions (pH 5.0), higher drug (64.3%-MH2 and 58.3%-MH1) release was noticed due to interactions between protonated carboxyl groups, PAA segments and TG and protonated DOX Saturation magnetizations of MGs were about 32.5 emu/g (32.5 Am^2^/kg) for MH2 and 23.9 emu/g (23.9 Am^2^/kg) for MH1, respectively	pH-responsive (5) meets the existing recommendations	Sayadnia et al. [168]
Skin cancer	Targeted drug release chemotherapy under magnetic stimulation	Alginate/DOX	Iron oxide superparamagnetic nanoparticles, gelatin particles	SCC7 cell line	Mice (6 weeks, 20 g) with SCC7 xenografted tumor (5 × 10^5^ cells/mouse)	In vitro: drug release under magnetic stimulation (0.3 T, 0.5 Hz for 120 s, every 30 min): the cumulative DOX release was about 60% within 120 min; the cell reduction was achieved for chemotherapy applied under magnetic field simulation In vivo: tumor growth was highly reduced in mice treated with MG under magnetic stimulation (0.3 T, 0.5 Hz for 2 min, one application per day). No important weight loss was observed	Magnetic stimulation’s importance	Kim et al. [169]
	Immunotherapy (PD-1) + MHT	Gelatinic–tannic acid-based gel/checkpoint inhibitor PD-1	Bimetallic magnetic nanoarchitectonics (Zn_0.35_Fe_0.65_) Fe_2_O_4_	B16F10 cell line	Female C57 mice with a B16F10 xenografted tumor (5 × 10^6^ cells/mouse) injected into the flank region	In vitro: absence of AMF (309 kHz, 40 kA/m): 90% viability, under AMF action: 60% viability. Analysis performed based on the CCK-8 assay In vivo: the inhibitory effect of the MG was analyzed on distant tumors and pulmonary metastases. A negative Ki-67 staining was achieved under AMF application, which proved the treatment efficiency obtained through the synergistic effect of pyrolysis and immunotherapy compared to the mice group that was treated only with MG, or MG loaded with PD-1 in the absence of AMF	*H* × *f* = 1.2 × 10^10^ A/(ms)	Wang et al. [182]
	Immunomodulator effect	Hyaluronic acid with borosilicate nanoparticles	Fe_3_O_4_	B16F10 cell line	Mouse animal model with B16F10 xeno-grafted tumor	In vitro: at the maximum concentration of MNPs (250 μg/mL), a reduced cell viability of less than 10% occurred. MG were incubated with interleukin 4 (IL-4)-pretreated M2 macrophages. An enhancement of M1 macrophages number concomitantly with an important decrease in M2 macrophages population was noticed In vivo: the MG group exhibited the lowest tumor fluorescence intensity and the smallest tumor volume. The mice’s body weight was maintained 10 days after surgery, indicating no signs of toxicity. The histopathological analysis revealed an increase in M1 macrophages compared to the M2 state	Macrophage state modulation	Quan et al. [183]
Lung cancer	Magnetic field-driven chemotherapy	Salecan-g-poly(vinylacetic acid-co-2-hydroxyethyl acrylate) [poly(VA-co-HEA)] copolymer/DOX	Fe_3_O_4_@Agarose	COS-7 and A549 cell lines	-	In vitro: DOX release was investigated under different pH values (pH of 7.4, 6.8, and 4.5 with/without a magnetic field (2200 G (0.22 T))) for endosomal and lysosomal microenvironment analysis. *M*_s_ of MG was about 19.8 emu/g (19.8 Am^2^/kg) at 15 kOe (1.2 MA/m). The DOX release was higher at pH 4.5 than at the others. The cumulative release of DOX could reach 76% after 64 h at acidic pH compared to normal tissue pH (pH 7.4—16.9%; pH 6.8–10.5%)	pH-responsive (4.5) meets the existing recommendations	Hu et al. [170]
Bone cancer	Remotely stimulated drug release under a low-frequency alternating magnetic field (LAMF)	Chitosan loaded with DOX (commercial name Adriamycin) and rifampicin (RFP)	Iron oxide MNPs	MG-63 cell line	-	In vitro: absence of LAMF: about 40% percent of DOX was released within 2 h, and after 4 h a continuous drug release was noticed. Regarding RFP, almost 60% percent of RFP was released within 60 h and the RFP emission was observed even after 100 h LAMF (60 Hz, 15 min ON, 15 min OFF, 1 h): LAMF for 1 h, the cumulative release percentage of DOX increased by 67.2% (from 37.5% to 62.6%); LAMF accelerated the RFP release	LAMF application importance	Wang et al. [76]
Head and neck cancer	Targeting delivery and multi-stimuli release of chemotherapeutic drug	DNA nanogel made via rolling circle amplification, an enzymatic polymerization of the DNA chain, and in situ coating of the MNPs based on physical crosslinking procedure/DOX	Amino-modified Fe_3_O_4_ nanoparticle (Fe_3_O_4_@SiO_2_-NH_2_) used as a core with a shell of DNA nanogel	U87MG cell line	-	In vitro: DOX, as a cationic molecule, can be linked to negatively charged DNA molecules. Firstly, the drug release of the magnetic DNA@DOX nanogel was tested at various temperatures and physiological pH levels. The release rate increased directly proportional to temperature and was 13% (25 °C), 25% (37 °C), and 59%(50 °C). Secondly, the pH influence was investigated. The release amount of DOX at 37 °C within 5 h was 25% (pH of 7.4), 31% (pH of 6.0), 38% (pH of 5.0), respectively. Thirdly, the presence of the nuclease was analyzed. The release amount of DOX at 37 °C (5 h) increased directly proportionally with DNase I concentration, up to 68% at 560 U/mL DNase I. The U87MG cells internalized the developed magnetic DNA nanogel	pH-responsive (5) meets the existing recommendations	Yao et al. [171]
Kidney cancer (nephroblastoma)	Targeted drug release	Silk fibroin/DOX	Fe_3_O_4_ (25 μg of powder)	Laboratory-developed human fibroblast cells (isolated from the foreskin) and Wilms’ tumor cells	-	In vitro: absence of electromagnetic field application: the DOX release profile was in a direct relationship with viscosity and porosity. At lower MG viscosity, the drug moved faster. Regarding the porosity, adding the MNPs increased the porosity, which facilitated the DOX release Electromagnetic field application: AMF (5 mT), static magnetic fields (SMF1 0.18 T and SMF2 0.28 T): reduced DOX release compared to EMF absence: the release of DOX from MG decreased with increasing the SMF magnetic flux density value. DOX release was diminished in the presence of AMF compared with SMF1 and SMF2 applications	Comparison between the effects of AMF and SMF application	Haghighattalab et al. [121]
Potential large B-cell lymphoma	MHT + Chemodynamic therapy (Pt) + Immunomodulation	Poly(lactic-co-glycolic) PLGA-polyethylene glycol (PEG)-PLGA	Fe_3_O_4_@Dimercaptosuccinic acid (DMSA)@Platinum (Pt)	A10 cell line	-	In vitro: Flow cytometry revealed an initial increase in ROS levels at 12 h, followed by a reduction at 48 h. This phenomenon was accompanied by a low cell viability and a high cell apoptosis	ROS importance, *H* × *f* = 3.5 × 10^9^ A/(ms) meets the existing recommendations	Yan et al. [184]

ER—estrogen receptor, PR—progesterone receptor, HER2—human epidermal growth factor receptor 2, EGFR—epidermal growth factor receptor, NIPAM—poly(N-isopropylacrylamide), DEAEMA—2-(diethylamino)ethyl methacrylate, TMSPMA—3-(trimethoxysilyl)propyl methacrylate, HPMC—hydroxypropyl methyl cellulose, L929—mouse fibroblast cell line [131], MCF-7 human breast cancer cell line [131], HUVEC—human umbilical vein endothelial cells [131], MDAMB231—highly aggressive, invasive, poorly differentiated triple-negative breast cancer (TNPC) [131], HT-29 human colorectal carcinoma cell line [131], H22—murine hepatocellular carcinoma cell line [131], VX2—anaplastic squamous cell carcinoma derived from Shope papillomavirus infection in rabbit [131], BT549—human breast cancer cell line [131], Erβ2—estrogen receptor β, Y5R—neuropeptide Y Y5 receptor, TRPC4—transient Receptor Potential Canonical 4, AR—androgen receptor, HEK293T—human embryonic cell line [131], PBS—phosphate buffered saline solution, HeLa—immortalized cervical cancer cell line [131], RAW 264.7—murine macrophage cell line [131], 4T1—murine mammary carcinoma cell line derived from a BALB/c mouse—model for triple-negative breast cancer [131], RPMI-1640—Roswell Park Memorial Institute medium 1640, NIR—near—infrared light, LoVo—adenocarcinoma, colorectal Duke’s type C (grade IV) human cell line [131], SCC7—squamous cell carcinoma 7 [131], A549—adenocarcinomic human alveolar basal epithelial cells [131], DOX—doxorubicin hydrochloride, COS-7—immortalized cell line derived from African green monkey kidney cells [131], DNA—deoxyribonucleic acid, U87MG—human glioma cell line [131], B16F10—mouse melanoma cell line [131], A10—myoblast-like cell from the rat thoracic aorta [131], CCK-8 assay—cell counting kit.

### 4.4. Magnetic Hydrogel Toxicity

Hydrogel toxicity is of utmost importance when medical applications are discussed. Its main toxicity issues are due to MNPs’ incorporation and are related to oxidative stress, DNA changes, and inflammatory reactions. In this direction, the investigation of Nowak-Jary and Machnicka [186] analyzed many literature studies and established that a certain value for MNP size of about 10 nm was considered to have reduced toxicity and oxidative stress [187,188]. On the other hand, some investigations [189,190,191,192] proved that larger particles induced important effects such as cell death on MCF-7 cell line, human brain endothelial cells, murine macrophages and lymphocytes, osteosarcoma cells, and A549 cells based on reactive oxidative species. In cancer therapy, ROS are considered beneficial due to the fact that they can increase the cell death mechanisms based on ferroptosis or other phenomena, but they must not induce damage to the neighboring healthy tissue.

Another concern related to the MNPs’ use consists of their genotoxic effects. Refs [193] and [194] showed that direct interactions were established between MNPs and DNA in the nucleus, being linked to damage in genetic material functions such as nucleotide oxidation and modifying the transcription and replication processes. These unwanted effects could be controlled by using biocompatible coatings for MNPs, as well as an adequate concentration that reduces the DNA alteration. Other side effects that were in a direct relationship with cell modifications were considered the cytoskeleton disruption [195], cell membrane disruption [196], and cell cycle alteration [197].

For MG, one must also consider the inflammatory response that is usually associated with an increased level of immune system cell number, heat shock proteins, and proinflammatory cytokines (IL-6, IL-1a, IL-1β, TNF-α). It is understood that for a cancer treatment approach, this fact could be considered a positive one, because the inflammatory reaction at the tumor site could trigger a body response that fights against the neoplasm cells, but, as in most cases, the so-called “MNPs’ dose” makes a difference. To sustain this affirmation, systemic and organ-toxic effects should be investigated. It was found that in some cases, MNPs exhibited a given neurotoxicity for murine animal models [198,199,200], but another investigation [201] proved a paradoxical effect concomitantly with a reduced ROS emission. Regarding the respiratory system, mild inflammatory responses [202] of the lung parenchyma and a moderate intralveolar septal thickening [203] were reported for the rat animal model. Some MNPs accumulate in the liver, spleen, and lymph nodes, and biocompatible coating application reduced the amount of this side effect [204,205]. Other organ behavior-related toxicity issues are exhaustively presented in [17,18,186].

**Figure 5:**
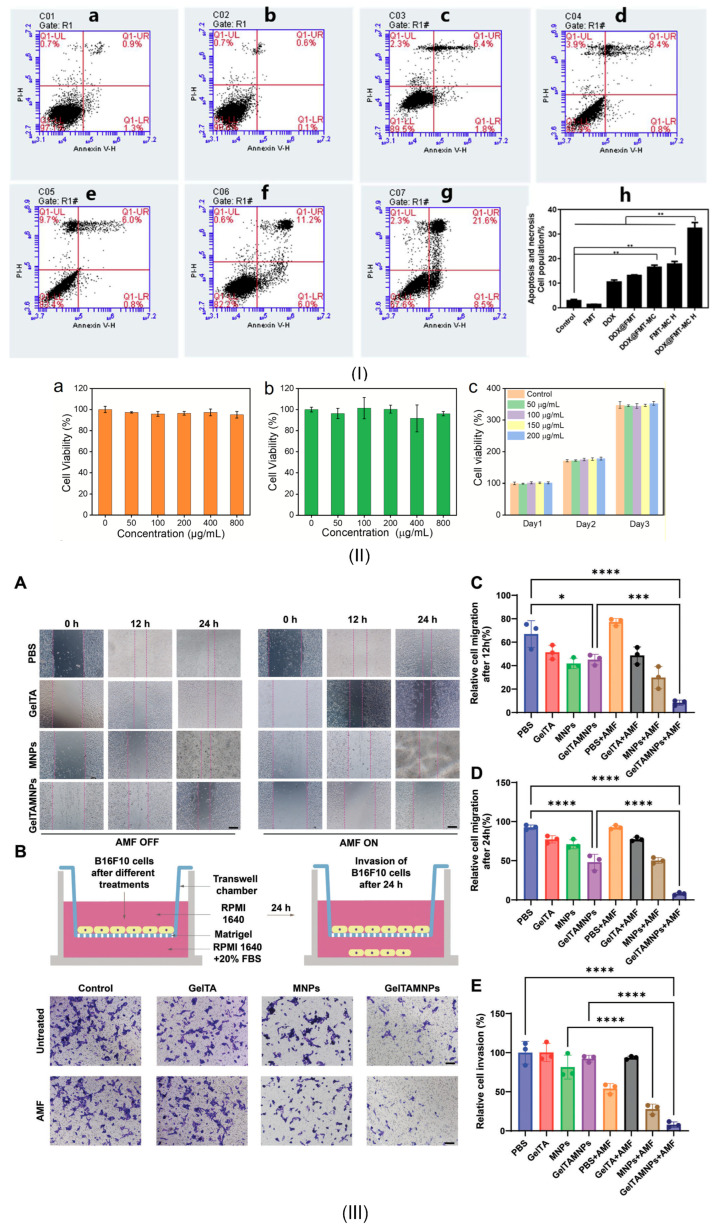
In vitro experimental investigations obtained in the case of different MGs used as potential oncological treatment: MHT + Chemotherapy—(**I**) Flow cytometric analysis on death of various HT-29 cell groups using Annexin V-FITC/PI under different conditions: (**a**) Control, (**b**) Ferumoxytol (FMT), (**c**) Doxorubicin (DOX), (**d**) DOX@FMT, (**e**) DOX@FMT-medical chitosan (MC), (**f**) FMT-MC with magnetic hyperthermia (H), (**g**) DOX@FMT-MC with H, (**h**) analysis regarding cell death (necrosis and apoptosis) for each condition (** *p* < 0.01). Reprinted from [143]. Copyright 2025 Elsevier; MHT + Chemotherapy—(**II**) Cell viability of NIH-3T3 incubated with different concentrations of: (**a**) gelatin nanoparticles (GNPs), (**b**) magnetic montmorillonites (MMTs), (**c**) magnetic colloidal gels (MCGs). Reprinted from [115]; Immunotherapy + MHT—(**III**) Anti-migratory and anti-invasiveness in vitro attributes of gelatin–tannic acid hydrogel with bimetallic nanoalloy (GelTAMNPs): (**A**) Wound-healing assay pictures of B16F10 cells at different time intervals (0 h, 12 h, 24 h) (PBS—phosphate buffered saline solution, MNPs—magnetic nanoparticles, GelTA—gelatin–tannic acid hydrogel) (scalebar 100 μm), (**B**) Schematic representation of the in vitro experiment in the AMF presence or absence (FBS—fetal bovine serum), (**C**,**D**) Cell migration to enclose the artificial gap after 12 h and 24 h incubation time, (**E**) Relative cell invasion rates after 24 h (* *p* < 0.05, *** *p* < 0.001, **** *p* < 0.0001) Reprinted from [182].

**Figure 6:**
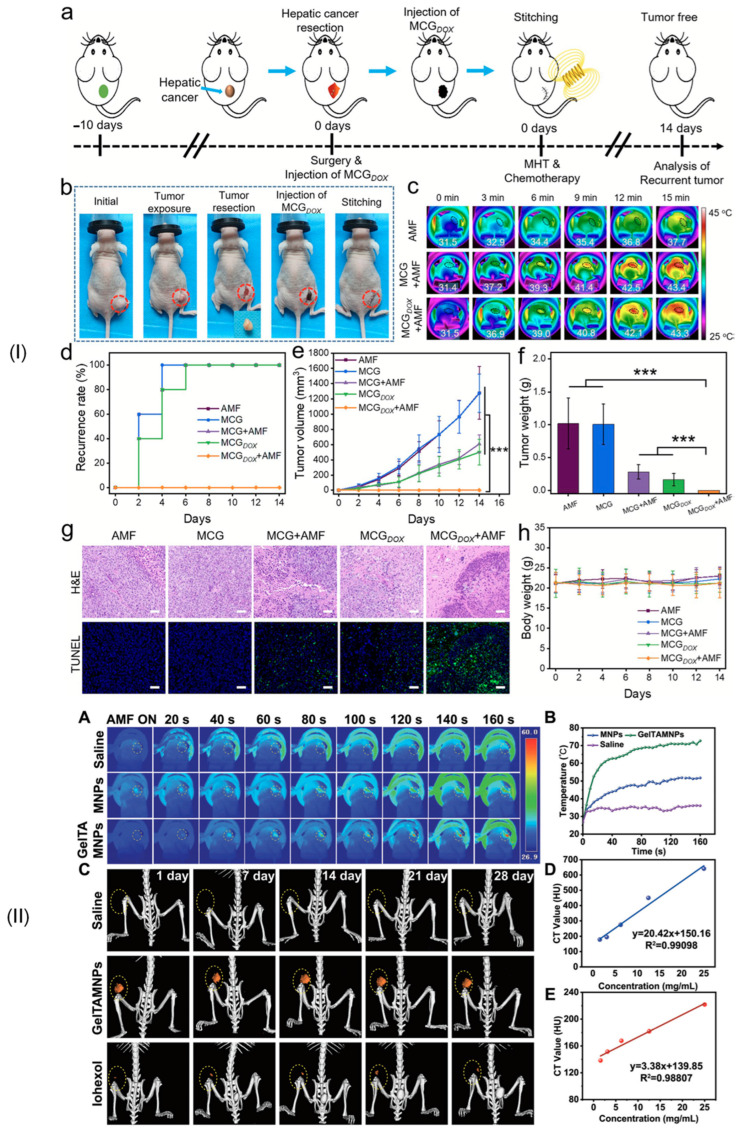
In vivo experimental studies obtained in the case of different MGs used as potential oncological treatment: MHT + Chemotherapy (**I**) Combined oncological treatment to prevent tumor recurrence (**a**) schematic diagram of treatment, (**b**) surgical images, (**c**) Infrared thermal images of post-surgical tumor site with AMF, (**d**) Recurrence rate of tumor after various treatments, (**e**) tumor volume variation, (**f**) tumor weight values after different treatment approaches, (**g**) H&E and TUNEL staining analysis after 14 days for each treatment (scalebar 50 μm), (**h**) animal model body weight during 14 days (*** *p* < 0.001). Reprinted from [115]; Immunotherapy + MHT—(**II**) In vivo multimodal imaging performance: (**A**) Real-time images of tumor xenografted mice after saline, bilayer MNPs, and GelTAMNPs, under AMF irradiation up to 160 s, (**B**) Temperature versus time at the tumor site, (**C**) Computer tomograph (CT) images at different time intervals, CT value standard curves for (**D**) GelTAMNPs treatment and (**E**) Iohexol contrast agent application. Reprinted from [182].

To minimize the above-mentioned risks, surface modifications, controlled release of active substances, and targeted delivery must be considered, as will be presented in the future trends section. Figure 5 and Figure 6 present a selection of experimental data obtained after in vitro (Figure 5) and in vivo (Figure 6) investigations in the case of magnetic hydrogels as a potential cure for cancer.

Another important aspect to consider is the clearance kinetics of MGs, which are usually analyzed using magnetic resonance imaging (MRI). The MG is administered by injection and can be monitored to reveal the mechanisms underlying its elimination from the body. The clearance rate and distribution traces of MG depend on material properties and delivery methods. In some cases, magnetic susceptibility artifacts at interfaces with natural tissue, arising from local magnetic field distortions, can generate magnetic resonance imaging artifacts. Other problems, such as ghosting and blurring, are direct results of the MG motion, as seen in patient blood flow. To address these drawbacks, adjustments to MRI acquisition parameters and the use of MRI-compatible MG could be implemented.

The toxicity of magnetic hydrogels could enhance cancer cell death by improving treatment efficacy, as shown in Table 4. A selection of examples was extracted from the literature to highlight the positive toxicity issue in a direct relationship with ROS generation, including ferroptosis, cytotoxicity, genotoxicity, and DNA modifications.

## 5. Conclusions, Challenges, Future Trends, and Limitations

**Figure 7:**
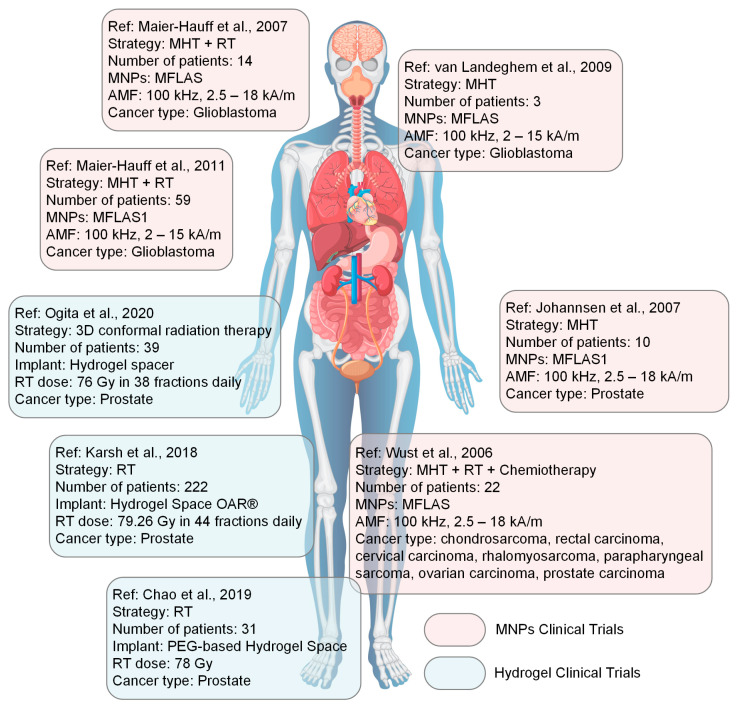
Main clinical trials performed on various patient groups with different oncological diseases based on MNPs (Maier-Hauff et al., 2007 [209], Maier-Hauff et al., 2011 [206], van Landeghem et al., 2009 [211], Johannsen et al., 2007 [210], Wust et al., 2006 [208]) and hydrogel spacers (Ogita et al., 2020 [212], Karsh et al., 2018 [213], Chao et al., 2019 [214]). Abbreviations: MNPs: MFLAS1, MagForce, 12 nm aminosilane coated Fe_3_O_4_ nanoparticles; MFLAS, MagForce, 15 nm aminosilane coated Fe_3_O_4_ nanoparticles, AMF—alternative magnetic field, RT—radiotherapy, MHT—magnetic hyperthermia. This figure was generated using images assembled from www.freepik.com, accessed on 10 September 2025.

The most important challenge regarding magnetic hydrogels consists of their clinical translation. Although some clinical trials describe the MNPs’ use for glioblastoma multiforme, prostate carcinoma, chondrosarcoma, epithelial carcinoma, ovarian carcinoma, cervical carcinoma, and rectal carcinoma [206,207,208,209,210,211], and a hydrogel spacer for prostate cancer [212,213,214] there is no clinical trial that investigates the magnetic hydrogel use (Figure 7). MGs could be considered as a dedicated platform and future approach in cancer therapy because they combine different features and could be used in multi-strategy oncological therapies with localized application and better patient outcomes. However, although the pre-clinical studies presented in this review underline treatment efficiency, supplementary improvements are necessary. Firstly, the quality and biocompatibility of the MNPs should be investigated. Secondly, reproducible experimental protocols are necessary, and standardized features should be adopted. In addition, biodegradable MGs must be used in conjunction with reduced toxicity for the byproducts. Overall, MG should be considered as a future therapy because of their potential to be magnetically controlled to release different chemotherapeutic drugs as well as to initiate MHT effects, or they can be applied for immunotherapeutic response.

In comparison with the drawbacks associated with the MNPs’ administration [25], incorporating them into a polymeric hydrogel matrix prevents MNPs from agglomeration and leakage. In this way, their toxic accumulation into different organs such as the liver and spleen [215,216] and related side effects, is hindered. This fact makes them a safer strategy in cancer therapy compared to the classical MNPs’ suspensions. Regarding MHT approaches, a reduced saturation magnetization is expected due to particle immobilization into the hydrogel matrix, but the application of high-frequency magnetic fields could easily overcome this. In addition, MHT treatments can be combined with chemotherapy or immunotherapy, offering precise localized and sustained drug release as well as a proper immune response from the host body. These innovative treatments exhibit a high potential for different cancer types as presented in the review paper.

As future trends, adding different types of nanoparticles to the MG, such as carbon nanotubes, bioglasses, and silicon, could enhance the hydrogel’s mechanical properties and add supplementary features such as absorption of radiation in different frequency domains. In addition, magnetic hyperthermia should be enhanced to offer a better outcome and could be easily combined with the magnetic imaging technique if gadolinium-based MNPs are involved. Another feature investigation should be devoted to the development of different biocompatible coating layers [217,218,219] for MNPs to decrease their toxicity and accumulation, as presented in Section 4.4. Limitations such as residual catalysts used for drug covalent bindings in theragnostic and the potential breakage of coatings or vehicle materials must also be considered. Another important problem consists of MNPs’ toxicity, as previously discussed. In Ref [220], it was proved that the non-toxicity level for superparamagnetic MNPs was established at about 100 μg/mL. At the same time, Ref. [221] analyzed the median lethal MNP dose in the hamster animal model and found it to be between 300 ÷ 600 mg_Fe_/kg body weight for uncoated MNPs and 2000 ÷ 6000 mg_Fe_/kg body weight for coated MNPs.

Another future trend could be linked to MG’s use in immunotherapy due to magnetic materials’ versatility and biocompatibility. Some studies were presented in this review paper that underlined the effect of MGs in inducing an immune response in the presence or absence of an external magnetic field. However, some limitations must be considered, such as poor stability of some immunomodulatory drugs and their thermal fluctuation when MHT is applied as a complementary approach to immunotherapy. To address the drawbacks mentioned above and to keep under control the toxicity and clearance of the MNPs, their size, saturation magnetization, and coatings must be adapted in good accordance with the medical applications, and a not “one size fits all” approach should be considered.

**Figure 8:**
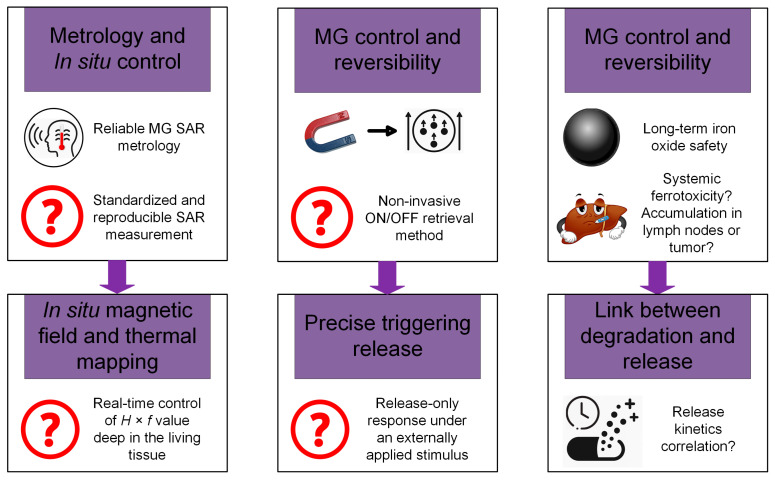
Key outstanding question box emphasizes the most important issues that must be solved before clinical trials initiation.

In our opinion, if the scientific gaps related to MNPs’ toxicity and quantity are overcome, magnetic hydrogels are a good candidate for clinical trials and could be used in different cancer treatments with a localized focus and reduced side effects if some important questions are answered and solved (Figure 8).

Taking into consideration all the literature studies analyzed in the review, a decision table (Table 5) that summarizes the “pros” and “cons” of the MG synthesis methods, in vivo investigations, and clinical translation is presented below.

A “Key Outstanding Questions” box is presented in Figure 8. The topic of magnetic hydrogels must address questions related to the reliability and standardization of heating metrology, hydrogel control and reversibility, and biological safety for long-term use. The two identified questions in a direct relationship with SAR metrology are: how can a standardized and reproducible method be employed for SAR measurement in different media, considering the effects of non-linear magnetic fields and MNP aggregation, and how could it be possible to control the value of *H* × *f* product in real time by making experimental determination in situ within living tissue. Another important problem that should be addressed before clinical trials start concerns MG control and reversibility: if the active drug is “turning off” or the MG is removed, it must be possible to reverse the significant side effects that occur. The question is: which type of methods (i.e., non-invasive or minimally invasive ones) could be imagined for a controlled retrieval or a full and swift deactivation/reversal of the therapeutic effect of MGs? Another task must address precise triggering and the manufacturing of a temperature-sensitive gate on MGs to ensure a reversible, tight, and release-only response under the action of an external AMF or a specific pH of the environment. Regarding long-term safety, one can ask about the organ-specific clearance rates and long-term systemic outcomes of metallic ions in the lymph nodes or tumor zone. If iron oxide MNPs are used, Fe^2+^ or Fe^3+^ ions can enter completely via the ferritin or transferrin pathways in the natural iron cycle, or they can generate ferroptosis or other types of ROS when higher MNP concentrations are incorporated into hydrogels. The last question could be identified as how to establish a correlation between the degradation rate of the hydrogel matrix and the ion release kinetics with the desired oncological healing window to ensure the safety of the patient’s organs. All the above-mentioned questions should be answered through future studies before clinical trials on human patients start.

## Figures and Tables

**Table 4:** MNPs’ toxicity issues beneficial for oncological applications.

MNPs Core	Hydrodynamic Size	Coating	Dose Metric	Exposure to Outcomes	Remarks in Relation with Oncology	Ref.
Fe_3_O_4_	2.3, 4.2, and 9.3 nm	-	100 mg/kg	Decreased MNPs toxicity; an increase in ROS in serum and internal organs in the 2.3 nm MNPs group, (2.16, 1.61, 4.03, and 1.9 times higher than in the control group). ROS level in the 2.3 nm Fe_3_O_4_ group was the highest compared to other hydrodynamic size particles	The ultrasmall Fe_3_O_4_ with sizes of 2.3 and 4.2 nm was highly toxic, compared to the larger MNPs with little toxic effects (9.3 nm)	Wu et al. [187]
Iron oxide MNPs	10 and 30 nm	Polyethylene glycol (PEG)	10 mg/kg body weight MNPs; control group (PBS solution)	MNPs induced the production of IL-6 and tumor necrosis factor-α (TNF-α). The inflammatory reactions were size-dependent, demonstrating that 30 nm MNPs induced greater oxidative stress	Exposure to MNPs has enhanced Neutrophil migration and infiltration	Ying et al. [188]
Fe_3_O_4_	60 nm	Coated with biocompatible polymers (non-specified in the study)	15, 50, or 100 mg/kg body weight	The decrease in cell viability was linked to an increase in ROS generation as a function of MNPs dose	Used in conjunction with thymoquinone, the oxidative stress and genetic toxicity were attenuated	Ansari et al. [189]
γ-Fe_2_O_3_	15 nm	L-Glutamic acid	128 μg/mL gel, cell density 5 × 10^5^ cells/mL	A slight increase in DNA damage was noticed compared to the control group. ROS apparition was evidenced in in vitro study, and a maximum effect was achieved at the highest MNPs concentration	No genotoxicity effects were noticed in combination with reduced toxicity. Acellular and intracellular ROS were present with no DNA damage	Zhang et al. [191]
Fe_3_O_4_	12 nm	DMSA-coated MNPs	50 and 100 µg/mL	The transcription of the Id3 gene was significantly downregulated in different cell lines treated with two doses (50 and 100 µg/mL) of coated MNPs	The MNPs exerted a significant effect on the expression of Id genes	Zou et al. [194]
Fe_2_O_3_	14, 22, and 30 nm	-	12.5, 25, 50, 100, and 200 lg/mL gel	Genotoxicity and cytotoxicity tests have shown that MNPs are more toxic to cells compared to free ferric ions	The proteomic investigation proved cytoskeleton disruption and an important influence on cell division when MNPs are used as a potential tumor treatment	Askari et al. [195]
Carob-leaf synthesized iron oxide nanoparticles	15.63 ± 2.38 nm	-	10 mg/kg	The hippocampus and striatum of Wistar rats exhibited increased toxicity related to MNPs	A good potential for the carob-synthesized MNPs for the treatment of cerebral cancer was foreseen	Fahmy et al. [198]
Fe_3_O_4_	8 nm ÷ 10 nm	DMSA, PEG, PEG-Au	15 mM	ROS existence was evidenced in T cells in nerve concomitantly with activation of the inflammation (MMP-9, IL-1β) signaling pathways	The intraneural MNPs injection into the sciatic nerve in rats proved an enhanced toxicity with applicability in cancer therapy	Kim et al. [199]

**Table 5:** Decision analysis of the pros/cons of MG synthesis technologies, in vivo tests, and possible clinical translation.

Section	Pros	Cons	Recommendation for Clinical Translation
MG preparation methods and main attributes	Blending: fast and simple method (attribute—superparamagnetism); In situ: uniform distribution (attribute—biocompatibility); Ex situ: scalability and ease of processing (attribute—tunable properties); Grafting onto: no MNPs leakage, higher stability of magnetic properties (attribute—magnetic property stability)	Blending: MNPs aggregation and poor distribution; In situ: complex process requires a large number of precursors; Ex situ: MNPs non-uniform diffusion and aggregation; Grafting onto: time-consuming and challenging procedure	One should choose the synthesis method by considering the particularity of the oncological application
In vivo tests	MHT: good hyperthermia effects and possible chemoembolization; Drug delivery: improved drug delivery process and controlled release; Immunotherapy: good potential even in the absence of an external AMF	MHT: potential toxicity; Drug delivery: difficulty in drug release control; Immunotherapy: increased costs and unexpected immune reactions	The in vivo tests for MHT showed promising potential for cancer cell death, but the risks of some cellular damage must be considered in relation to MNP toxicity. When localized drug delivery is involved, reduced systemic side effects are achieved, but precise drug release control could be hard to conduct. Regarding immunotherapy, unexpected immune reactions may occur alongside MNPs’ toxicity.

## Data Availability

The original contributions presented in the study are included in the article, further inquiries can be directed to the corresponding author.
